# Evaluation of loci to predict ear morphology using two SNaPshot assays

**DOI:** 10.1007/s12024-022-00545-7

**Published:** 2022-11-19

**Authors:** Saadia Noreen, David Ballard, Tahir Mehmood, Arif Khan, Tanveer Khalid, Allah Rakha

**Affiliations:** 1 Department of Forensic Sciences, University of Health Sciences, Lahore, 54600 Pakistan; 2 King’s Forensics, King’s College London, Franklin-Wilkins Building, 150 Stamford Street, London, UK; 3 School of Natural Sciences (SNS), National University of Sciences and Technology (NUST), Islamabad, H-12 Pakistan; 4 Genomics Group, Faculty of Biosciences and Aquaculture, Nord University, 8049 Bodø, Norway; 5 Department of Human Genetics and Molecular Biology, University of Health Sciences, Lahore, 54600 Pakistan

**Keywords:** Ear morphology predictions, Forensic DNA phenotyping, Predictive DNA analysis, EVCs

## Abstract

Human ear morphology prediction with SNP-based genotypes is growing in forensic DNA phenotyping and is scarcely explored in Pakistan as a part of EVCs (externally visible characteristics). The ear morphology prediction assays with 21 SNPs were assessed for their potential utility in forensic identification of population. The SNaPshot™ multiplex chemistries, capillary electrophoresis methods and GeneMapper™ software were used for obtaining genotypic data. A total of 33 ear phenotypes were categorized with digital photographs of 300 volunteers. SHEsis software was applied to make LD plot. Ordinal and multinomial logistic regression was implemented for association testing. Multinomial logistic regression was executed to construct the prediction model in 90% training and 10% testing subjects. Several influential SNPs for ear phenotypic variation were found in association testing. The model based on genetic markers predicted ear phenotypes with moderate to good predictive accuracies demonstrated with the area under curve (AUC), sensitivity and specificity of predicted phenotypes. As an additional EVC, the estimated ear phenotypic profiles have the possibility of determining the human ear morphology differences in unknown biological samples found in crimes that do not result in a criminal database hit. Furthermore, this can help in facial reconstruction and act as an investigational lead.

**Supplementary Information:**

The online version contains supplementary material available at 10.1007/s12024-022-00545-7.

## Introduction

The externally visible traits of humans are complex, resulting from polygenic inheritance [1–3]. Human ear morphology is signified as a highly polymorphic and polygenic trait that exhibits continuous phenotypic distribution and serves as an important target in forensic DNA phenotyping studies [4]. The variability exists among phenotypes of lobe sizes and states, degree of ear protrusion and the difference in helix shape, tragus and antitragus morphology in each individual [5]. In forensics, external ear morphology has been used since Bertillon (1893) for personal identification from photographic images, videos, or ear prints in forensics [6]. An otoscopic forensic opinion has the status of scientific evidence which is admitted by Polish Courts [7]. Earlobe attachment can be highly useful in disaster victim verification [8, 9]. The medico-legal importance of the ear is due to its stable structure and rigidity in burnt bodies which further enables facial reconstruction [10]. Moreover, it is useful in the identification of drowning cases of mutilated faces [9, 11, 12].

Understanding the genetic aetiology is important for ear morphogenesis [13], forensic genetics [14] and diagnostics [15]. The first comprehensive study investigated the pinna trait in the Latin American population and identified seven loci for variations in human ear morphology using genome-wide association studies (GWAS) [16]. Another GWAS for variant association with lobe attachment in multi-ethnic groups (Europeans, Americans cohorts) identified 49 significant loci associations [4]. The genetic variations like SNPs insertion-deletion variants, block substitution and inversion variants may cause amino acid substitutions which alter the functional property of the protein [3]. This results in morphological changes and distinct phenotypes [17].

Previously developed phenotyping assays used a variety of reported techniques to obtain genotypic data including TaqMan assays [18, 19], next-generation sequencing (NGS), Ion Ampliseq technology [20] and whole-genome sequencing (WGS) [21]. However, whole-genome sequencing is an expensive technique and not suitable for the specific traits of interest involving limited genes. Multiplex analyses coupled with the mini sequencing technique offer a targeted approach for retrieval of specific phenotypes of interest [22–25]. The phenotypic variation in population caused by genetic variation must be added to modelling parameters [17]. Regression analyses are performed to model the structure (identify the pattern) seen within the dataset following odd ratios [26].

Several phenotyping methods with the multiplex genetic panels and prediction models have been proposed, for example, IrisPlex [27], HIrisPlex [28] and HIrisPlex-S [29]. Furthermore, progress has been made in inferring height [30], baldness [31] [32], freckles [20], hair thickness [33], age [34] and facial morphology from biological samples [35, 36]. Forensic DNA phenotyping (FDP) is the prediction of these externally visible characteristics (EVCs) from DNA traces [37–39]. The importance of forensic DNA analysis for criminal investigation is quite evident in the Zainab Murder case [40, 41]. It was confirmed through DNA testing when the 814th sample of suspects showed similarity with the reference sample in the database. In the absence of reference DNA, a DNA phenotyping study can be useful in narrowing down the pool of suspects and can potentially provide more details about the appearance of individuals than eyewitnesses can. It is used as an intelligence tool rather than to confirm individual identity [42].

In Pakistan, only one study is available focusing on the genetic determination of lobe attachment ear phenotype for Southern Punjab subjects [43]. Another study was found regarding the DNA-based prediction of eye colour in the Swat population [44]. No published data is available for other phenotypes of the ear. Much attention is paid to the diagnostic and genetics of hearing loss in Pakistan [45]. Whereas multiplex panels for EVCs prediction are often tested majorly in Europeans [46, 47], Eurasians Americans [48] and Koreans [49]. The utility of forensic DNA phenotyping is in its infancy in Pakistan. The frequency of ear morphological characteristics is well documented [14, 50–52].

To fill this gap, the ear phenotypes from a specific combination of genotypes are predicted in the Pakistan population. The study aims to improve the reliability of ear morphology prediction by harnessing three hundred individuals; thirty-three predicted categories from twenty-one significant genetic predictors from genes (*MRPS22*, *TBX15*, *EDAR*, *SH3RF3*, *TGOLN2*, *SP5*, *TF binding site*, *LOC107985447*, *SLC4A1PP1*, *LRBA*, *XPNPEP1*, *FLJ20021*, *GCC2*, *WDR3*, *LOC100287225*, *FOXL2*, *GPR126*, *LOC153910*, Antisense to *MYO3b*, *SULT1C2P1*) in previous GWAS were selected [4, 16].

## Methodology

### Human ear phenotypes and study cohort

The ear trait phenotypes were assessed with slight modifications in the previous study [14]. The ear trait was classified as (1) lobe size (small, medium, large); (2) lobe attachment (attached lobe, intermediate attachment, free; (3) antitragus (absent, average, prominent), (4) tragus size (absent, average, prominent), (5) posterior helix rolling (under folded, partial folded and over folded), (6) superior helix rolling (under folded, partial folded and over folded), (7) antihelix folding (under folded, partial, over folded), (8) antihelix superior crus (flat, intermediate and extended), (9) Darwin tubercle (absent, degree of presence and prominent), (10) crus helix expression (less prominent, prominent and extended) and (11) ear protrusion (small, medium and large) as shown in (Fig. 2).

A Nikon D5600 camera was used to photograph each ear along with the individual’s head in the Frankfort horizontal plane described by Meijerman et al. [53]. Phenotypes were assessed by high-quality photographs and closely observing the individual ear. The approval of this study was obtained from the ethical review committee of the University of Health Sciences, Lahore, Pakistan. Healthy males and females of age 18–40 years without ear abnormalities were considered in the study. DNA was extracted with an in-house standard protocol of phenol–chloroform isoamyl alcohol [54], and quantitative analysis was performed using Qubit 3 Fluorimeter with a double-stranded DNA broad range assay kit (Thermo Fisher Scientific) according to the manufacturer’s directions [55].

### Selection of targeted DNA variants

Genes and their common genetic variants were selected through a systematic literature search [4, 16]. It included twelve intronic (rs10212419, rs17023457, rs13397666, rs7567615, rs2080401, rs1960918, rs3818285, rs9866054, rs263156, rs260674, rs10192049), three intergenic (rs868157, rs1619249, rs1879495), three regulatory (rs7873690, rs6845263, rs10923574), one missense (rs3827760), one 3′UTR (rs7428) and one 5′ UTR (rs2378113) variant. Common genetic variants in regulatory or coding regions of a candidate gene with functional relevance assessed in silico were given high priority during selection. SNPs were assessed by the 1000 Genome Project Phase 3 allele frequencies in the Punjabis in Lahore (PJL) sub-population and were selected for genotyping analysis.

### SNP genotyping assay

Primer 3 plus was used to design 21 primer pairs and their respective single-base extension primers using the default parameters of the software program, targeting similar melting temperatures of 60 °C and similar GC contents. Primer sequences are detailed in Table 1 along with final PCR and SBE primer concentrations for both multiplexes [56]. The melting temperature and amplicon size were analysed in silico on the UCSC genome browser [57]. The potential performance of multiplex PCR primers was screened on Autodimer [58] to detect any hairpin and primer dimer formation. Both forward and reverse single-base extension primers were designed, and either one of them was added to the final multiplex system. Poly T-tails have been added to the 5′ end of the SBE primers to ensure complete capillary electrophoresis separation between the SBE products of multiplexes. Optimization of all primers was performed using gradient PCR. Multiplex PCR was performed in a 10-µl final reaction volume containing 1 × Qiagen PCR Multiplex Mix (Hilden, Germany), primer concentrations as specified in Table 1 and 5 ng of DNA. Thermal cycling was performed on a Veriti 96 well thermocycler (Applied Biosystems). The multiplex PCR conditions were as follows: 95 °C for 15 min, 30 cycles of 95 °C for 30 s, 60 °C for 90 s, 72 °C for 60 s and the final extension at 60 °C for 30 min. For removal of unincorporated primers and dNTPs, 3 µl of amplified product was purified with 1 µl Exosap (ExoproStart™) at 37 °C for 1 h and 75 °C for 15 min. Before performing a multiplex extension reaction, all SBE primers were verified for their proper working efficiency by executing the singleplex extension reaction with the corresponding template. The multiplex single-base assay reaction was prepared with final concentrations of 1 × SNaPshot™ ready mix (Thermo Fisher), SBE primer concentrations as stated in Table 1 and 1 µl of purified PCR product, in a Veriti 96-well thermocycler (Applied Biosystems) following thermocycling conditions: 96 °C for 2 min, 25 cycles of 96 °C for 10 s, 50 °C for 5 s and 60 °C for 30 s. The extension product was purified by the addition of 1 µl of SAP enzyme (Applied Biosystems), followed by incubation for 70 min at 37 °C and 20 min at 72 °C.

**Table 1:** SNP markers included in the Ear-Plex system for ear morphology prediction ordered according to prediction rank with molecular details and genotyping

Assay position	SNP ID	Chr number and region	Gene	Major allele	Minor allele	PCR primer sequences	Primer conc. (µM)	Product size	SBE primer sequence, length of SBE primer, and direction	Detected allele	SBE primer conc. (µM)
Plex1_1	rs10212419	3, intronic	MRPS22	CC	TT	F-CTTTGGGCTCAACCCGACTA R-TATGTGGAATGGGCTCTCCC	0.3	235	TTTTTTTTTTGCACACGTAGTATCTTGTATAACC, 34, R	T/C	0.2
Plex1_2	rs17023457	1, intronic	TBX15	TT	CC	F-TGGAGACTCTGAGACAACCTGA R-CCCACTCCTCACCAGAAACT	0.3	296	TTTTTTTTTTTTTGATACCGACCACTAACTAATCAACA, 38, F	T/C	0.2
Plex1 _3	rs13397666	2, intronic	EDAR	AA	GG	F-CAGGTCTGAACCGTAGCCAG R-CAGAGATGGCCTGAACCTCC	0.3	188	TTTTTTTTTTTTTTTTTTTTATAGGTCGGCGAGGTTCC, 38, F	A/G	0.2
Plex1 _4	rs7567615	2, intronic	SH3RF3	GG	AA	F-CTGTGAGGTCAACTGAGCGG R-CCCACAATGACAGCCACCTT	0.3	164	TTTTTTTTTTTTTTTTTTTTCCAACGATCAGAAAATAAACCC, 42, R	A/G	0.2
Plex1 _5	rs3827760	2, missense	EDAR	TT		F-AGAGTTGCATGCCGTCTGTC R-CCACGGAGCTGCCATTTGAT	0.3	159	TTTTTTTTTTTTTTTTTTTCACGTACAACTCTGAGAAGGCTG, 38, R	T/C	0.2
Plex1 _6	rs7428	2, 3′UTR	TGOLN2	TT	CC	F-TCAAACATGAAGTCTGGTGCATT R-ACCCCTGTTAGGAAGGTTGG	0.3	253	TTTTTTTTTTTTTTTTTTTTTTGCTTACTGGCAGTTTGACATACTA, 46, F	T/C	0.2
Plex1 _7	rs868157	1, intergenic	TF binding site LOC107985447	TT	GG	F-AGCCCTTGAATGAGGGTTGG R-GGGGGCTTGCACATCATAGA	0.3	229	TTTTTTTTTTTTTTTTTTTTTTATTATCTACCATACCAAAACTATGAGCT, 50, F	G/T	0.2
Plex1 _8	rs2080401	2, intronic	SP5	AA	CC	F-TAGTAGAGTAGCCCACAGA R-CTGGTCTTGAACTCCTGA	0.3	122	TTTTTTTTTTTTTTTTTTTTTTTTTGCAACTAGTAGAGTAGCCCACAGAA, 50, F	A/C	0.2
Plex1 _9	rs7873690	9, regulatory region	SLC4A1PP1	CC	TT	F-TTCCGTTGAAGGGTGCTGTA R-CCCTGAAACTGGAACAGAGCC	0.3	300	TTTTTTTTTTTTTTTTTTTTTTTTTTTTTTTTTTTTTCAGGGGAATCCCAGGAG, 54, F	T/C	0.2
Plex 1_10	rs1960918	4, intronic	LRBA	TT	CC	F-AACAAGAAACCAAGAACCCAAATA R-TCCTTCTTCCTGTCTGTCCTCTTA	0.3	254	TTTTTTTTTTTTTTTTTTTTTTTTTTTTTTTTTGAGATAATTGAGTGAATCTCGGTAA, 58, F	T/C	0.2
Plex2_1	rs3818285	10, intronic	XPNPEP1	AA	GG	F-GAACAGAGTCACAACTGGGCTA R-ACCTTATTGACTCGGGTGCT	0.4	215	TTTTTTTTTTTTTTAAGGTGGACAGCTGAGCTCC, 34, R	G/A	0.4
Plex2_2	rs6845263	7 regulatory region	FLJ20021	CC	TT	F-GCACCTCATCACTCTCTGCC R-AGGTTAGAAAAACTAACCCAGACT	0.4	285	TTTTTTTTTTCATCTGTATGTGTGCTGTGTTTGA, 34, F	T/C	0.4
Plex2 _3	rs2378113	2, 5′ UTR	GCC2	AA	GG	F-TTTTAGTGTGCGCAATCGCC R-AGCCCACAGATCAGAATCCC	0.4	208	TTTTTTTTATAAAGCAGTCTAAGAAGGTTTATATAGTG, 38, F	G/A	0.4
Plex2_4	rs10923574	1, regulatory region	WDR3	AA	CC	F-ACCCTATGAAAAGAGCATGTAGT R-AATCACGTAGACTGAGGGGA	0.4	263	TTTTTTTTTTTTTTTAACAGCCTTTTCAAGAAATACCTATTA, 42, F	A/C	0.4
Plex2 _5	rs1619249	2, intergenic	LOC100287225	TT	CC	F-CTTGATCTCCTGACCTCTT R-GTGGACTTTACATTTACTCTGA	0.4	84	TTTTTTTTTTTTTTTTTTTTTGTGGGCGGATAGGAGGC, 38, R	T/C	0.4
Plex2 _6	rs9866054	3, intronic	MRPS22, FOXL2	GG	AA	F-TTGAGGGCTTCTCTTGTGGC R-CCCACTGTCTTAAAGTAGCCCATT	0.4	109	TTTTTTTTTTTTTTTTTTTTTTTTAGCTGTTTTCTAGGCTGGATTG, 46, F	G/A	0.2
Plex2 _7	rs263156	6, intronic	GPR126, LOC153910	CC	AA	F-CAAAGGCCCATGCAGCTACT R-TTGGAAGGCACATCAACCAC	0.4	181	TTTTTTTTTTTTTTTTTTTTTTTTTTTCTCATCTACCCTATCATTCCACC, 50, R	A/C	0.2
Plex2_8	rs260674	2, intronic	EDAR	AA	GG	F-ACTCAAAACCGAGTGTCCCG R-TGAACCCCGCCAATGTCCTA	0.4	121	TTTTTTTTTTTTTTTTTTTTTTTTTTTTTTTTTTTGCTTTGGTTACGTCTGCCC, 54, F	G/A	0.1
Plex2 _9	rs10192049	2, intronic	Antisense to MYO3b	AA	GG	F-TCGTGGCAAGTTACGTGTGTA R-AATGCTTGGTGCACGGTAGG	0.4	281	TTTTTTTTTTTTTTTTTTTTTTTTTTTTTTTTTTAGTATCAGTCCATATGCCTTCACA, 58, R	G/A	0.4
Plex2_10	rs13427222	2, intronic	EDAR	AA	GG	F-GGCCTGATGGTTCGGAGTTA R-AAGGAGAG TAGCGCTGGGT	0.4	277	TTTTTTTTTTTTTTTTTTTTTTTTTTTTTTTTTTTTTTTTTTTTTCCAGCACCTTGCCTCCC, 62, R	A/G	0.4
Plex2_11	rs1879495	11, intergenic	SULT1C2P1	CC	AA	F-AAGTGACCTCCTGGACTTGG R-GCACCAGCAGGGGAAAGTA	0.4	299	TTTTTTTTTTTTTTTTTTTTTTTTTTTTTTTTTTTTTTTTTTTGGGTAGAACTGGAACAAAATCTT, 66, R	A/C	0.4

### Capillary electrophoresis and allele calling

The purified extension product (1 µl) was mixed with 10 µl Hi-Di formamide and 0.4 µl Genescan-120 Liz size standard and run on a 3130xl Genetic analyser (Applied Biosystem) after rapid heating of the reaction mix at 100 °C for 2 min and cooling for 2 min. The analyser has POP-7 as the sieving polymer, on a 36-cm capillary length under an injection voltage of 2.5 kV for 10 s and with a running time of 500 s at 60 °C using the default run module and E5 dye set. Allele calling and analysis of results were performed with GeneMapper™ ID software version 3.1.

### Statistical analysis

The output files generated through SNaPshot™ were analysed to assess levels of association between phenotype and genetic variation across all individuals typed.

#### Population analysis

All variant data were tested for Hardy–Weinberg expectations using the HWE calculator of Micheal H. Court’s (2005–2008) online calculator Excel-based HWE test and SNPstats (https://www.snpstats.net/start.htm). Linkage disequilibrium testing was performed with the online software SHEsis [59].

#### Association testing

To predict the probability of an ordered outcome of lobe sizes, tragus size, antitragus sizes, posterior and superior helix rolling, antihelix folding, antihelix superior crus, Darwin tubercle and crus helix expression, ordinal logistic regression was applied. The multinomial logit was performed on phenotypes of lobe states and ear protrusions being not in the order form. The multiple SNP association testing was performed using R programming through the multinomial regression. For each phenotype, one multinomial logistic regression or ordinal regression was applied, whatever is fitted. The significance regression coefficient of the respective genotype, i.e., SNP, was described by a Wald statistics-based *p*-value, with the threshold of 0.05. For better interpretation, the fitted model transforms the regression coefficients into the odds ratio. An odds ratio (OR) measures the effect of SNPs over the respective phenotype. An odds ratio equal to one indicates the change in SNP level in genotype has no effect on the phenotype, and odds ratio greater or below than one indicates the change in SNP level in genotype has an effect on the phenotype. 95% confidence intervals (CIs) and *p*-values were calculated for minor allele classifications. The dependent variable was coded as “1” for phenotype category 1 and “2” for phenotype 2 and 3 for phenotype 3 category.

#### Prediction modelling

The established sets of significant associated SNPs with phenotype were used for prediction modelling not all 21 SNPs. Prediction modelling was performed with R programming. The number of model parameters, *p*, must be such that: *p* ≤ min (n1, n2, n3)/10 where ni = number of observed phenotypes within each category (*i* = 1, 2, 3) and *p* = number of markers multiplied by the number of genotypes minus one. So, for 21 bi-allelic SNPs, each with 3 possible genotypes (two homozygotes and a heterozygote), separate testing and training set was employed to avoid model overfitting. Tenfold cross-validation was also used where data was split into a training dataset and testing dataset. And the trained model was also tested on test data. Ninety per cent of samples was called as ‘known group’ or training sets in which phenotypes were known and 10% samples were used in the testing set also known as ‘blind sample’ in which phenotype was not known. The performance of the fitted ordinal and multinomial regression model using the area under the receiver operating characteristic (ROC) curves was evaluated for final prediction accuracy on the dataset. The AUC basically can be considered as the probability that the test correctly identifies the phenotype. It is the integral of ROC curves ranging from 0 to 1. Additionally, the sensitivity of the model, specificity, negative predictive value (NPV), positive predictive value (PPV) and maximal probability approach was assessed. The threshold of probability for ear phenotypes prediction was tested ranging from *p*-value > 0.05 to > 0.09.

## Results

### Population data

**Fig. 1:**
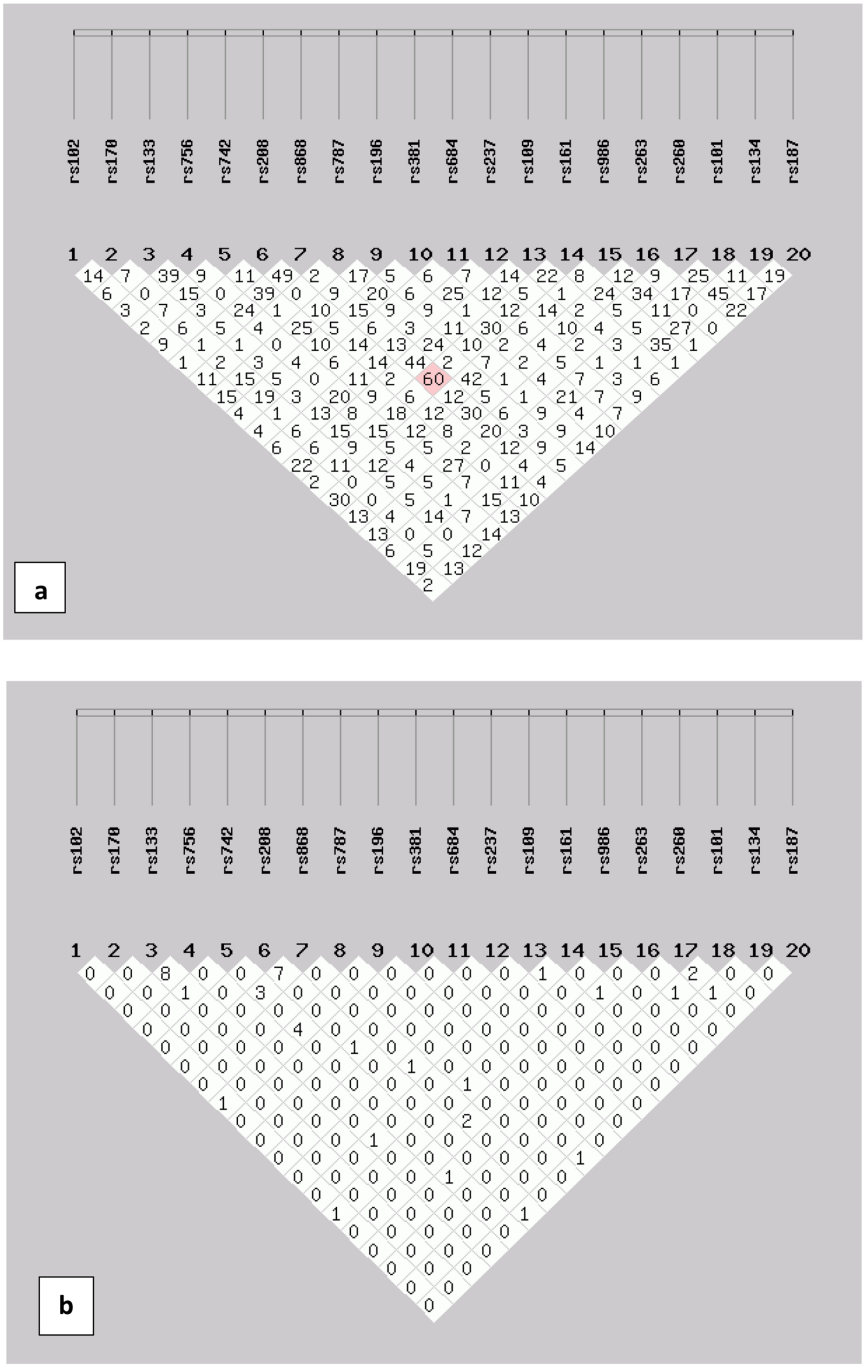

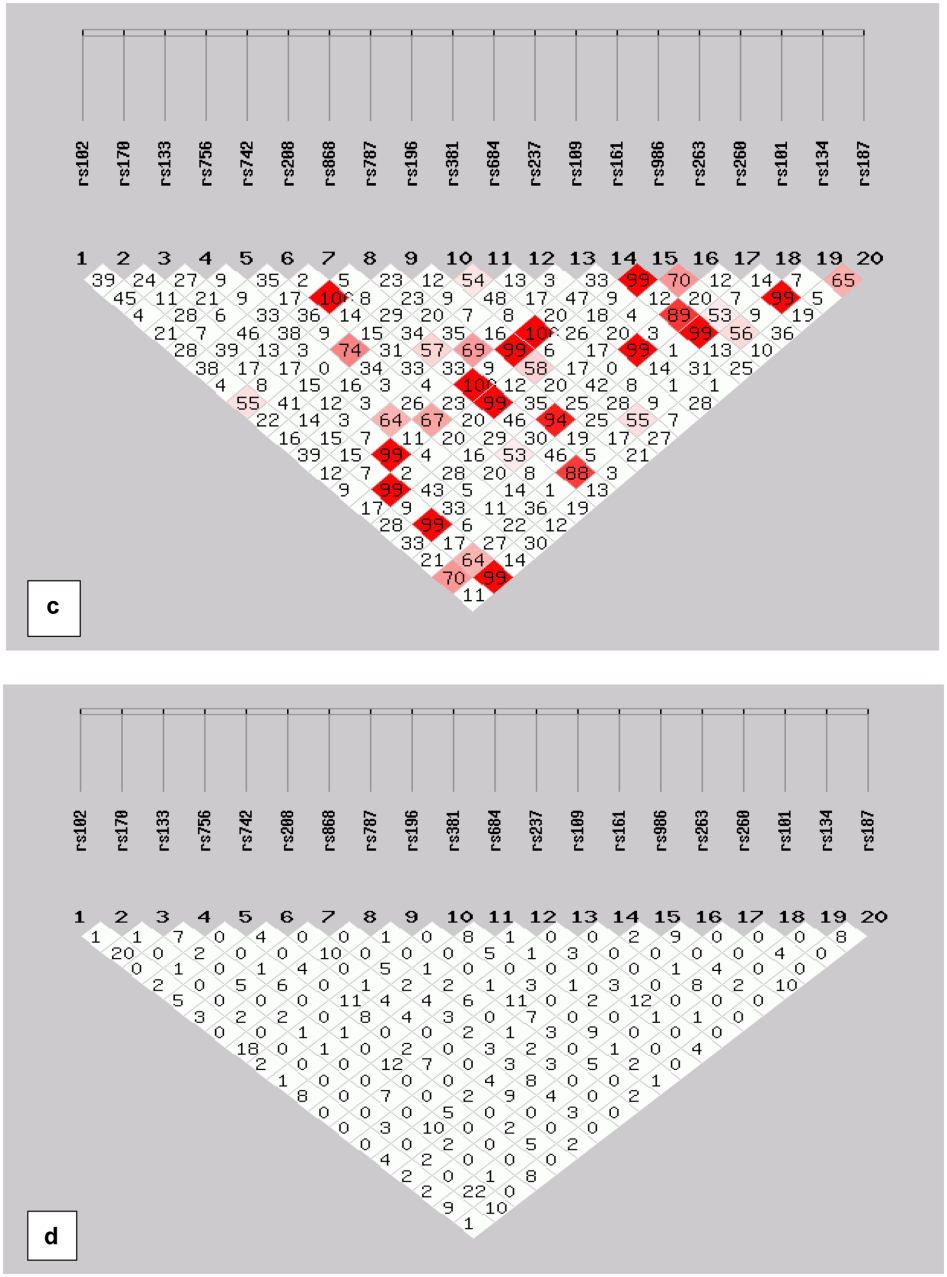
Linkage disequilibrium plots (LD) plot in all subjects. This figure shows the LD value in D (**a**) and *r*^2^ (**b**) between each SNP for controls. LD values in D (**c**) for cases and *r*.^2^ are shown in (**d**) between each SNP. Each diamond contains an LD value between the two SNPs that face each of the upper sides of the diamond. The redder the diamond, the higher the LD value. **a**, **b**, **d** indicate that LD is not detected for SNPs in controls and cases (SHEsis Software ver. online)

**Fig. 2:**
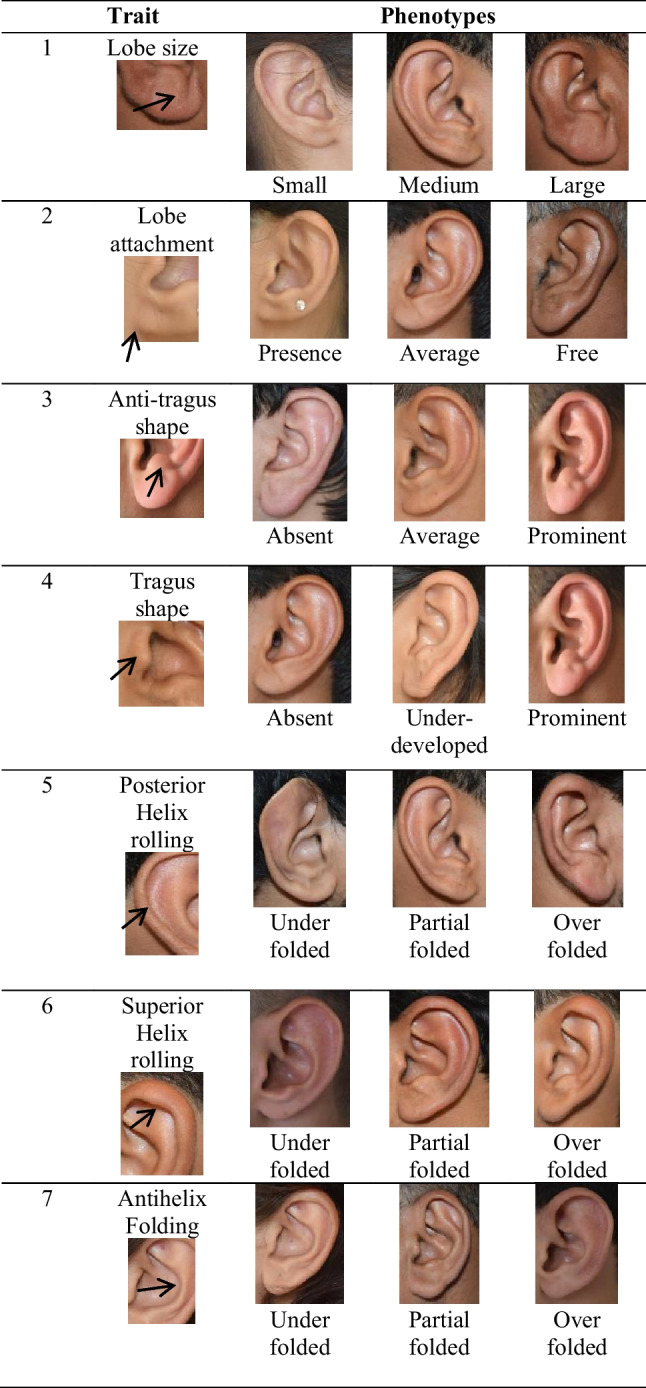

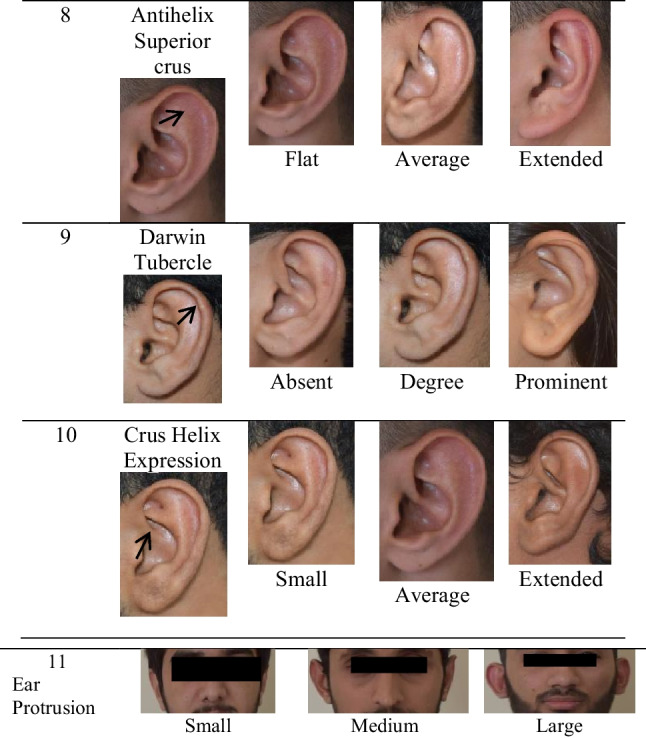
Ear phenotypes. The ear trait was classified as 1 lobe size (small, medium, large); 2 lobe attachment (attached lobe, intermediate attachment, free; 3 antitragus ( absent, average, prominent); 4 tragus size (absent, average, prominent); 5 posterior helix rolling (under folded, partial folded and over folded); 6 superior helix rolling (under folded, partial folded and over folded); 7 antihelix folding (under folded, partial, over folded); 8 antihelix superior crus (flat, average and extended); 9 Darwin tubercle (absent, degree of presence and prominent); 10 crus helix expression (small, prominent and extended); 11 ear protrusion (small, medium and large)

The percentage distribution of phenotypes is shown in Supplementary Fig. S1. The association testing was performed on all SNPs in order to draw the all-possible information in pilot-scale preliminary work. One rare SNP marker (rs3827760) was excluded at the level of statistical analyses (monomorphic in our dataset). Deviation from Hardy–Weinberg equilibrium was noted for few SNPs shown in Table S2 as the *p*-values < 0.05 were not consistent with HWE. Details of LD analysis are shown in Fig. 1. All the SNPs were not in Linkage disequilibrium. The ear traits and phenotypes are shown in Fig. 2.

### SNaPshot™ multiplex SNP genotyping assay and screening of genotypes

**Fig. 3:**
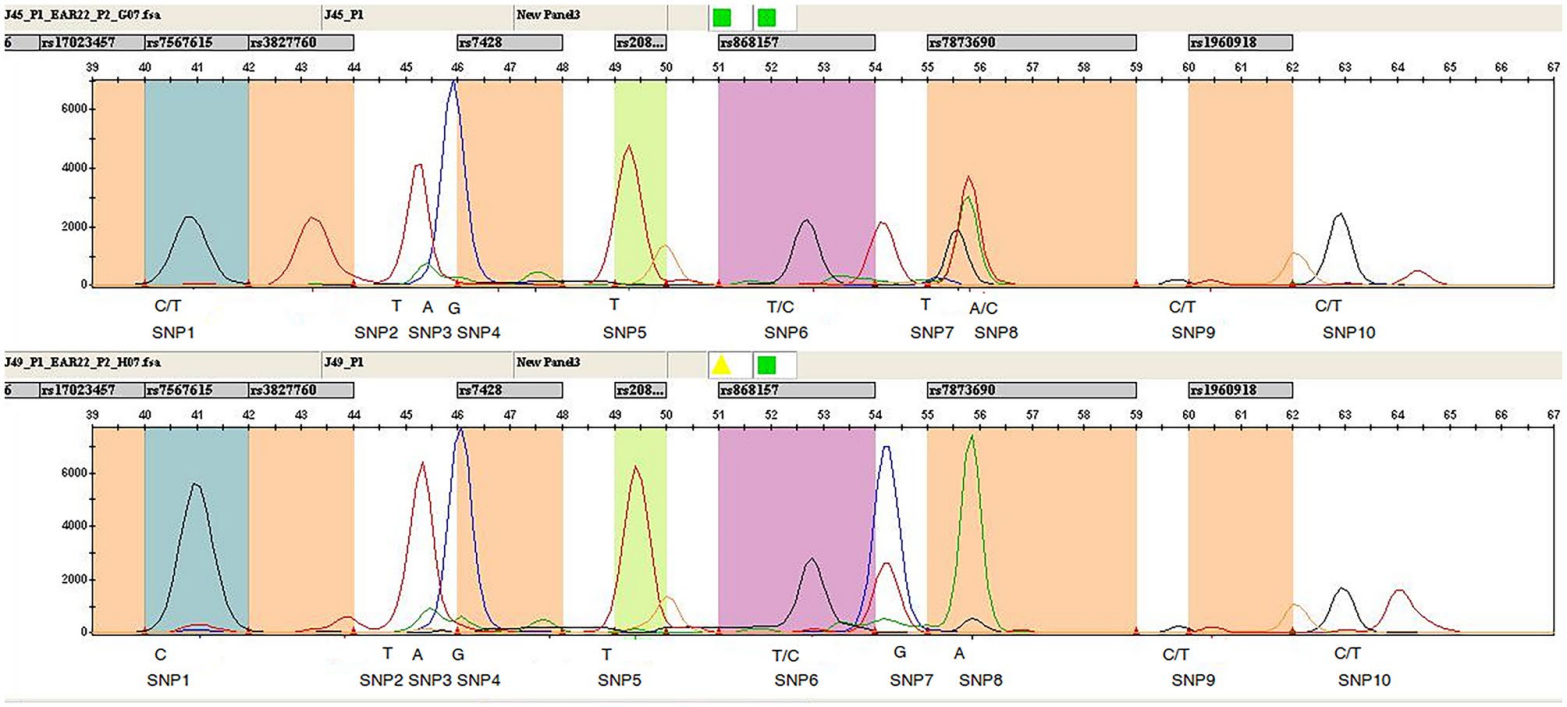
SNP genotyping electropherogram analysis in Plex-1. 10 SNPs generated a customized report with genotype results (including size, height, peak area). The vertical-coloured boxes are bins created automatically by the software using an extension product created with SNaPshot Kit. Each bin defines the minimum and maximum allowable size for each allele and identifies each peak and assigns the corresponding allele. Polymorphisms were identified based on peak size and colour. The C/T heterozygote allele of rs10212419 (SNP1), the homozygote allele T/T for rs17023457 (SNP2), the homozygote A/A allele of rs13397666 (SNP3), the homozygote G/G allele of rs7567615 (SNP 4), the homozygote T/T allele of rs3827760 (SNP5), the heterozygote C/T allele of rs7428 (SNP6), the T/T homozygote allele (SNP7), the A/A homozygote allele of rs868157 (SNP 8), the C/T heterozygote of rs7873690 (SNP9), and the C/T heterozygote allele of rs1960918 (SNP10) are shown in the electropherogram of the above sample. The C/C homozygote allele (SNP1), the homozygote allele T/T (SNP2), the homozygote A/A (SNP3), the homozygote G/G allele of (SNP 4), the homozygote T/T allele of (SNP5), the heterozygote C/T allele of rs7428 (SNP6), the G/G Homozygote allele (SNP7), the A/A homozygote allele of (SNP 8), the C/T heterozygote of (SNP9), the C/T heterozygote allele of (SNP10) are shown in the electropherogram of the sample below

**Fig. 4:**
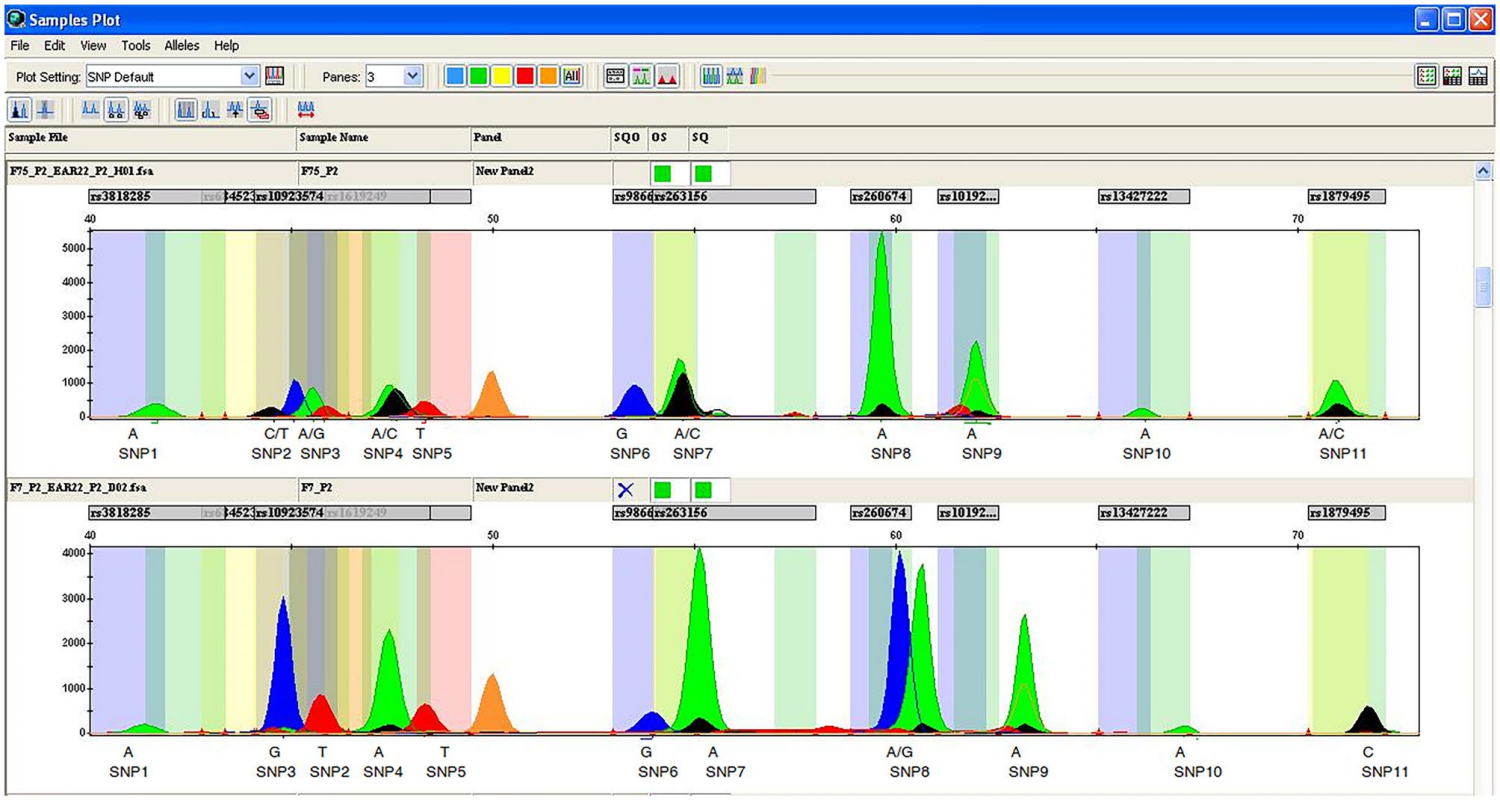
SNP genotyping electropherogram analysis in Plex-2. Samples were run with an ABI 3130xl Genetic Analyzer, using POP-7 on a 36-cm capillary length array. The electropherograms correspond to extended genotype showing 11 fragments. Peak size and colour vary according to polymorphism. The size (bp) of the primer with combined nucleotide is shown on the x-axis. RFU (relative fluorescence unit) of the peak is presented on the *y*-axis. The A/A homozygote allele of rs3818285 (SNP1), the heterozygote allele C/T for rs6845263 (SNP2), the heterozygote A/G allele of rs2378113 (SNP3), the heterozygote A/C allele of rs10923574 (SNP 4), the homozygote T/T allele of rs1619249 (SNP5), homozygote G/G allele of rs9866054 (SNP6), the A/C heterozygote allele of rs263156(SNP7), the A/A homozygote allele of rs260674s (SNP 8), the A/A homozygote allele of rs10192049 (SNP9), the A/A homozygote allele of rs13427222 (SNP10), the heterozygote allele A/C of rs1879495( SNP11) are shown in the electropherogram of above the sample. The A/A homozygote allele of rs3818285(SNP1), the homozygote allele T for rs6845263 (SNP2), the heterozygote G/G allele of rs2378113 (SNP3), the homozygote A/A allele of rs10923574 (SNP 4), the homozygote T/T allele of rs1619249 (SNP5), the homozygote G/G allele of rs9866054 (SNP6), the A/A homozygote allele of rs263156(SNP7), the A/G heterozygote allele of rs260674s (SNP 8), the A/A homozygote allele of rs10192049 (SNP9), the A/A homozygote allele of rs13427222 (SNP10), and the homozygote allele C/C of rs1879495( SNP11) are shown in the electropherogram in the below sample

The two genotyping assays were based on the principle of multiplex PCR followed by multiplex single-base extension assays using SNaPshot™ chemistry: Plex-1 assay encompassed of 10 SNPs whereas Plex-2 included 11 SNPs. Amplicons were designed to be 300 bp or smaller in length. According to the quality of amplicons, primer concentrations and annealing temperatures were optimized. Both the PCR and SBE multiplexes were optimized to achieve the balanced Plex-1 and Plex-2 SNP genotype profile (Figs. 3 and 4). The activity of SBE primer was verified by executing a single Plex extension reaction with a corresponding template PCR product. DNA input in assays was 5 ng. All expected peaks were detected, sized properly with accurate genotyped with uniform strength as shown in Figs. 3 and 4. Each peak was fragmented into genotypes and was interpreted following the peak(s) present at that site, with a single peak indicating homozygous genotype for that allele and double peaks indicating a heterozygote genotype for that SNP. Peaks with a relative fluorescence unit (RFU) value below 50 were excluded.

### SNP associations testing in Punjab population

As demonstrated in Table 2, the highest statistical significance was obtained for the seven SNPs including rs17023457, rs13397666, rs1960918, rs1619249, rs9866054, rs13427222 and rs1878495, explaining the variation in lobe size. The individuals’ genotype changed from CC to TT in rs17023457, 3.049 times (*p*-value = 0.045) more likely to have large lobe size. The individual genotype changed from GG to AG in rs13397666 with 0.454 times (*p*-value = 0.043), from CC to CT in rs1960918 with 0.466 time (*p*-value = 0.042), from CC to CT in rs1619249 with 0.180 times (*p*-value = 0.031), from AA to AG in rs9866054 with 0.376 times (*p*-value = 0.041), from GG to AG in rs13427222 with 0.150 times (*p*-value = 0.001), from GG to AA in rs3427222 with 0.221 times (*p*-value = 0.009) and from AA to CC in rs1878495 with 0.457 times (*p*-value = 0.044) less likely to have large lobe size.

**Table 2:** SNP association testing: *p-*value and odd ratio from ordinal and multinomial logistic regression performed on all SNPs to reveal their association with ear morphological traits

			*B*	Std. error	*p-*value	Odds ratio
Lobe size	Small		−7.665	1.8989	.000	.100
	Medium		−4.471	1.8518	.016	.214
	Large		References			
	rs17023457	TT	1.115	.5556	.045	3.049
		CT	1.087	.6193	.079	2.965
		CC	References			
	rs13397666	AA	−.477	.4120	.247	.621
		AG	−.789	.3942	.043	.454
		GG	References			
	rs1960918	TT	−.387	.4297	.368	.679
		CT	−.765	.3758	.042	.466
		CC	References			
	rs1619249	TT	−1.074	.7516	.153	.342
		CT	−1.716	.7965	.031	.180
		CC	References			
	rs9866054	GG	−.790	.4227	.062	.454
		AG	−.979	.5011	.041	.376
		AA	References			
	rs13427222	AA	−1.509	.5779	.009	.221
		AG	−1.900	.5955	.001	.150
		GG	References			
	rs1878495	CC	−.784	.4071	.044	.457
		AC	−.274	.4000	.493	.760
		AA	References			
Lobe attachment	Presence		−.339	1.9358	.861	.712
	Intermediate		2.334	1.9441	.049	5.318
	Free		References			
	rs7873690	CC	.649	.4071	.111	1.914
		CT	.976	.4860	.045	2.654
		TT	References			
	rs1960918	TT	−.470	.4226	.266	.625
		CT	−.706	.3789	.042	.493
		CC	References			
	rs1619249	TT	3.194	.9180	.001	4.376
		CT	2.961	.9452	.002	1.919
		CC	References			
	rs13427222	AA	−1.400	.6092	.022	.246
		AG	−1.390	.6148	.024	.249
		GG	References			
Anti-tragus size	Absent		.535	1.7502	.760	1.708
	Average		2.808	1.7572	.049	7.579
	Prominent		References			
	rs868157	TT	1.641	.8342	.049	5.159
		GT	1.086	.8747	.214	2.963
		GG	References			
	rs7873690	CC	−.841	.3868	.030	.431
		CT	−1.115	.4580	.015	.328
		TT	References			
	rs13427222	AA	1.017	.5084	.045	2.765
		AG	.612	.5176	.237	1.844
		GG	References			
Tragus size	Absent		.932	1.8039	.605	2.540
	Average		4.112	1.8261	.024	6.083
	Prominent		References			
	rs17023457	TT	1.155	.5747	.044	3.175
		CT	1.148	.6366	.071	3.151
		CC	References			
	rs868157	TT	1.655	.8096	.041	5.235
		GT	1.312	.8559	.125	3.714
		GG	References			
	rs7428	TT	−.839	.3622	.021	.432
		CT	−.683	.3191	.032	.505
		CC	References			
	rs7873690	CC	.579	.3989	.147	1.784
		CT	.897	.4783	.041	2.452
		TT	References			
	rs684523	CC	.533	.3095	.085	1.704
		CT	.653	.3153	.038	1.922
		TT	References			
	rs1619249	TT	1.724	.7839	.028	5.609
		CT	1.369	.8121	.092	3.931
		CC	References			
	rs263156	CC	.398	.3492	.254	1.489
		AC	−.683	.3464	.049	.505
		AA	References			
Superior helix rolling	Under folded		−.402	1.8691	.830	.669
	Partial folded		3.424	1.8775	.048	7.691
	Over folded		References			
	rs13397666	AA	.221	.4244	.603	1.247
		AG	.806	.4134	.041	2.240
		GG	References			
	rs7567615	GG	.898	.3844	.020	2.454
		AG	.283	.4915	.565	1.327
		AA	References			
Posterior helix rolling	Under folded		.326	1.7224	.850	1.386
	Partial folded		2.981	1.7317	.045	5.716
	Over folded		References			
	rs7428	TT	−.839	.3622	.021	.432
		CT	−.683	.3191	.032	.505
		CC	References			
	rs684523	CC	.533	.3095	.085	1.704
		CT	.653	.3153	.038	1.922
		TT	References			
	rs1619249	TT	1.724	.7839	.028	5.609
		CT	1.369	.8121	.092	3.931
		CC	References			
	rs263156	CC	.398	.3492	.254	1.489
		AC	−.683	.3464	.049	.505
		AA	References			
Antihelix folding	Under folded		−.699	1.8591	.707	.497
	Partial folded		2.986	1.8709	.031	4.806
	Over folded		References			
	rs2080401	AA	.474	.4031	.239	1.607
		AC	.915	.3790	.016	2.496
		CC	References			
	rs260674	AA	−1.663	.9087	.047	.190
		AG	−1.511	.9323	.105	.221
		GG	References			
Antihelix superior crus	Flat		−6.903	1.9609	.000	.001
	Intermediate		−4.480	1.9352	.021	.011
	Extended		References			
	rs17023457	TT	−1.048	.6025	.082	.351
		CT	−1.462	.6622	.027	.232
		CC	References			
	rs7567615	GG	.780	.3618	.031	2.182
		AG	.635	.4815	.187	1.887
		AA	References			
	rs1960918	TT	1.230	.4330	.005	3.420
		CT	.842	.3729	.024	2.321
		CC	References			
	rs9866054	GG	−.933	.4727	.048	.393
		AG	−.790	.5549	.154	.454
		AA	References			
	rs10192049	AA	−.939	.4278	.028	.391
		AG	−.503	.4265	.238	.605
		GG	References			
	rs13427222	AA	−1.322	.6336	.037	.267
		AG	−1.348	.6419	.036	.260
		GG	References			
	rs1878495	CC	−.811	.4275	.048	.444
		AC	.014	.4231	.974	1.014
		AA	References			
Darwin tubercle	Absent		2.221	28.9650	.049	6.000
	Degree of Tubercle		2.587	28.9650	.999	1.076
	Prominent		References			
	rs13397666	AA	1.107	.7257	.127	3.026
		AG	1.244	.6863	.049	3.471
		GG	References			
	rs260674	AA	−2.081	.9516	.029	.125
		AG	−1.624	.9752	.096	.197
		GG	References			
		AA	0^b^			1
Crus helix expression	Less		−.171	1.6985	.920	.843
	Prominent		1.857	1.7030	.048	6.402
	Extended		References			
	rs7428	TT	−.695	.3582	.052	.499
		CT	−.639	.3055	.036	.528
		CC	References			
	rs2080401	AA	−.672	.3812	.048	.510
		AC	−.352	.3529	.319	.703
		CC	References			
	rs7873690	CC	−.741	.4060	.048	.476
		CT	−.802	.4749	.091	.449
		TT	References			
Ear protrusion	Under folded		−1.488	1.7271	.044	.226
	Partial folded		.861	1.7263	.618	2.366
	Over folded		References			
	rs263156	CC	−.639	.3487	.047	.528
		AC	−.273	.3401	.422	.761
		AA	References			
	rs1878495	CC	−.746	.3985	.041	.474
		AC	−.596	.3887	.125	.551
		AA	References			

Four genetic predictors (rs7873690, rs1960918, rs1619249, rs13427222) have shown significant association with the attached ear lobe. The individuals’ genotype changed from TT to CT in rs7873690 with OR = 2.654 times (*p*-value = 0.045), from CC to CT in rs1619249 with OR = 1.91 times (*p*-value = 0.002) and from CC to TT in rs1619249 with 4.376 times more likely to get free ear lobes. The individuals’ genotype change from CC to CT in rs1960918 is 0.493 times (*p*-value = 0.042), from GG to AG in rs13427222 is 0.249 times (*p*-value = 0.024) and from GG to AA in rs13427222 is 0.246 times (*p*-value = 0.022) less likely to have free ear lobes.

Three SNPs (rs868157, rs7873690, rs13427222) explain the variation in antitragus size. The individual genotype changed from GG to TT in rs868157, with 5.159 times (*p*-value = 0.049) and from GG to AA in rs13427222 with 2.76 times (*p*-value = 0.045) more likely to get prominent antitragus. The genotype change from TT to CT in rs7873690 is 0.328 times (*p*-value = 0.015) less likely to get prominent antitragus.

Seven genetic predictors (rs17023457, rs868157, rs7428, rs7873690, rs684523, rs1619249, rs263156) were significantly associated with tragus size. The individuals’ genotype change from CC to TT in rs17023457 is 3.175 times more likely (*p*-value = 0.044), from GG to TT in rs868157 5.235 times (*p*-value = 0.041), from TT to CT in rs7873690 2.452 times (*p*-value = 0.041), from TT to CT in rs684523 1.922 times (*p*-value = 0.038) and from CC to TT in rs1619249 5.609 times (*p*-value = 0.028) more likely to get prominent tragus. The individuals’ genotype change from CC to CT in rs7428 is 0.505 times (*p*-value = 0.032), from CC to TT in rs7428 0.432 times (*p*-value = 0.009) and from AA to AC in rs263156 0.505 times (*p*-value = 0.049) less likely to get prominent tragus.

The highest statistical significance was obtained for two SNP (rs13397666, rs7567615) which explains the variation in superior helix rolling. The subjects’ genotype change from GG to AG in rs13397666 is OR = 2.24 times (*p*-value = 0.041) and the genotype change from AA to GG in rs7567614 2.4 times (*p* value = 0.02) more likely to get over folded superior helix rolling.

Four genetic predictors (rs7428, rs684523, rs1619249, rs263156) were significantly associated with posterior helix rolling. The individual genotype alters from CC to CT in rs7428 which is 0.505 times (*p*-value = 0.032), from CC to TT in rs7428 which is 0.432 times (*p*-value = 0.021) and from AA to AC in rs26315 which is 0.505 times (*p*-value = 0.049) less likely to get over folded posterior helix rolling. The genotype changed from TT to CT in rs684523 making it 1.922 times (*p*-value = 0.038) and from CC to TT in rs1619249 making it 5.609 times (*p*-value = 0.028) more likely to get prominent posterior helix rolling.

Two SNPs (rs2080401, rs260674) were significantly associated with antihelix folding. The subject genotype change from CC to AC in rs2080401 with 2.496 times (*p*-value = 0.016) more likely to have over folded antihelix folding. The genotype change from GG to AA in SNP rs260674 is 0.190 times (*p*-value = 0.041) less likely to get prominent antihelix folding.

The highest statistical significance was obtained for seven SNPs (rs17023457, rs7567615, rs1960918, rs9866054, rs10192049, rs13427222, rs1878495) which explains variation in antihelix superior crus. The individual genotype change from AA to GG in rs7567615 is 2.182 times (*p*-value = 0.031) and from CC to TT in rs1960918 is 3.420 times (*p*-value = 0.005) more likely to have extended antihelix superior crus. The individual genotype change from CC to CT in rs17023457 is 0.232 times (*p*-value = 0.027), from AA to GG in rs9866054 0.393 times (*p*-value = 0.048), from GG to AG in SNP rs1342722 0.260 times (*p*-value = 0.238), from GG to AA in rs10192049 0.391 times (*p*-value = 0.028), from GG to AA in rs13427222 0.267 times (*p*-value = 0.037) and from AA to CC in rs1878495 0.444 times (*p*-value = 0.048) less likely to get prominent antihelix superior crus.

Two SNPs (rs13397666, rs260674) were significantly associated with Darwin tubercle. The individuals’ genotype change from GG to AG which is rs1339766 is 3.471 times (*p*-value = 0.049) more likely to have prominent Darwin tubercle. The genotype change from GG to AA in rs260674 is 0.125 times (*p*-value = 0.029) less likely to get prominent Darwin tubercle. Three genetic predictors (rs7428, rs2080401, rs7873690) were significantly associated with crus helix expression. The individuals genotype change from CC to CT in rs7428 is 0.528 times (*p*-value = 0.036), from CC to AA in rs2080401 is 0.510 times (*p*-value = 0.048) and from TT to CC in rs7873690 is 0.476 times (*p*-value = 0.048) less likely to get extended crus helix expression. Two SNPs (rs263156, rs1878495) were significantly associated with ear protrusion. The genotype changed from AA to CC in rs1878495, with 0.474 times (*p*-value = 0.041) and from AA to CC in rs263156 with 0.474 times (*p*-value = 0.041) less likely to get large protrusion. The complete details of Table 2 are discussed in the supplementary material of Table S2.

### Prediction modelling accuracy

**Fig. 5:**
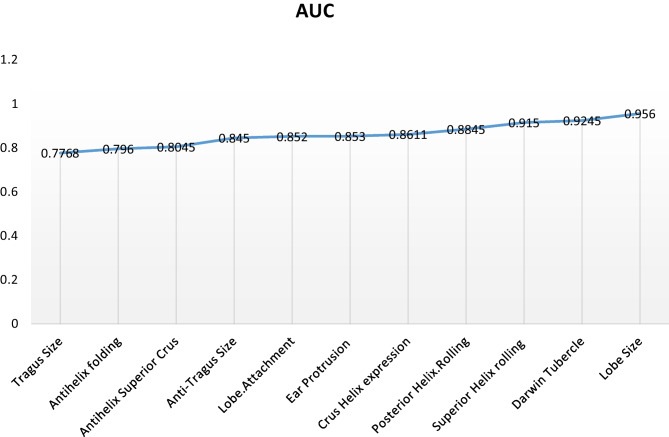
The area under the ROC curve for each fitted model represents the prediction of lobe size, and Darwin tubercle showed excellent predictions whose AUCs are 0.956 and 0.9245. The highly predictive accuracy obtained for superior helix rolling (0.915), posterior helix rolling (0.8845), crus helix expression (0.8611), ear protrusion (0.853), lobe attachment (0.852), antitragus size (0.845), and antihelix super crus (0.8045). The reasonably good predictive accuracies were obtained for antihelix folding (0.796) and tragus size 0.7768

The predictive model calculated probability of belonging to a particular class. A cutoff value was selected between 0 and 1, and if the calculated probability was over that threshold, the observation was assigned to the class. Overall excellent prediction accuracy of the multinomial model reached the value of AUC = 0.956 for lobe size, AUC = 0.9245 for Darwin tubercle, AUC = 0.915 for superior helix rolling and AUC = 0.8845 for superior helix rolling. The highest prediction accuracy of the model was obtained for posterior helix rolling AUC = 0.884, for crus helix expression AUC = 0.8611, for ear protrusion AUC = 0.853, for lobe attachment AUC = 0.852, for antitragus size AUC = 0.845 and for antihelix superior crus AUC = 0.8045. The reasonably good prediction accuracies were for antihelix folding AUC = 0.796 and tragus size = 0.7768 shown in Fig. 5.

Sensitivity is a proportion of true positive identified correctly. The highest sensitivity of the model was observed for medium lobe size (76.9%), free ear lobe (76.9%), absent antitragus (78.2%), large tragus (65.7%), under folded posterior helix rolling (76.25%), under folded superior helix rolling (61.75%), under folded antihelix folding (68.025%), extended antihelix superior crus (63.83%), absent Darwin tubercle (61.75%) and large crus helix (63.3%) individuals. The lowest sensitivity of the models was obtained for attached ear lobes (24.1%), average antitragus (19.4%), average tragus (37%), partial posterior helix rolling individuals (18.3%), partial folded superior helix rolling individuals (27.1%), medium crus helix (30.8%), average antihelix superior crus (33.7%), average Darwin tubercle individuals (27.1%), medium crus helix (30.8%) and medium protruding ear individuals (31.02%). Intermediate sensitivity was obtained for average attachment (56.8%), absent tragus (49.2%), over folded posterior helix (56.3%), over folded superior helix rolling (60.1%), over folded antihelix folding (57.3%), flat antihelix superior crus (53.38%), prominent Darwin tubercle (60.1%), small crus helix (56.8%) and flat ear (48.82%) individuals as shown in Table 3. The details are shown in the supplementary file Table S3.

**Table 3:** Accuracy of prediction modeling

Model	Phenotype	Sensitivity%	Specificity%	PPV%	NPV%
Multinomial	Small lobe size	56.8	81.5	62.3	77.8
	Medium lobe size	76.9	50.3	63	66.3
	Large lobe size	18.1	99.1	56.7	90.4
	Attached ear lobe	24.1	99.1	56.7	90.4
	Average attachment	56.8	81.5	62.3	77.8
	Free ear lobe	76.9	50.3	63	66.3
	Absent antitragus	78.2	51.6	64.3	67.6
	Average antitragus	19.4	95.8	58	91.7
	Large antitragus	58.1	82.8	63.6	79.1
	Absent tragus	49.2	73.95	59	76.95
	Average tragus	37	89.65	56.8	83.7
	Large tragus	65.7	64.85	61.3	71.1
	Under folded posterior helix	76.25	50.2	62.8	65.55
	Partial folded posterior helix rolling	18.3	96.8	54.65	90.65
	Over folded posterior helix rolling	56.3	81.1	61.6	77.5
	Underfolded superior helix rolling	61.75	61.375	60.15	70.225
	Partial folded superior helix rolling	27.1	93.725	54.05	86.65
	Over folded superior helix rolling	60.1	72.125	60.45	73.5
	Underfolded antihelix folding	68.025	55.0875	60.725	66.8625
	Partially folded antihelix folding	22.15	95.7625	52.675	88.125
	Over folded antihelix folding	57.3	75.7625	60.025	74.7
	Flat antihelix superior crus	53.38	69.76	59.38	74.71
	Average antihelix superior crus	33.7	91.01	55.88	84.68
	Extended antihelix superior crus	63.83	67.28	61.02	71.9
	Absent Darwin tubercle	61.75	61.38	60.15	70.23
	Average Darwin tubercle	27.1	93.73	54.05	86.65
	Prominent Darwin tubercle	60.1	72.13	60.45	73.5
	Small Crus helix expression	60.56	65.92	61.36	73.96
	Medium crus helix expression	30.68	91.71	58.09	86.96
	Large crus helix expression	63.3	72.01	62.66	74.5
	Flat ear	48.82	67.96	60.3	75.43
	Medium protrusion	31.02	90.11	58.02	85.19
	Large protrusion	64.51	66.97	62.33	72.78

However, on the contrary, the highest specificity large lobe size individuals (99.1%), attached ear lobes individuals (99.1%), average antitragus size (95.8%), average tragus size (89.65%), partial folded posterior helix rolling (96.8%,) partial folded superior helix rolling (93.725%) and partial folded antihelix folding prediction were recorded (95.7625%); average antihelix superior crus (91.01), average Darwin tubercle (93.73%) and medium crus helix were recorded (91.71%) and medium protrusion individuals (90.1%). Lowest specificity was observed for medium lobe size, 50.3% for free ear lobes, 51.6% for absent antitragus, 64.85% for large tragus, 50.2% for under folded posterior helix rolling, 61.375% for under folded superior helix rolling, 55.0875% for under folded antihelix folding, 67.28% for extended antihelix superior crus, 61.38% for absent Darwin tubercle, 65.92% for small crus helix expression and 66.97% for large protruding subjects as shown in Table 3.

## Discussion

Our phenotypic characteristics were comparable with previous studies [16, 52]. The Ear-Plex was designed on a similar pattern to IrisPlex and HIrisPlex-S [60] [29]. It was verified that all SBE primers worked correctly by executing a single Plex extension reaction with the corresponding template PCR product. This step was important when considering low-level peak height that may be susceptible to dropout when multiplexed as demonstrated in previous studies [61]. DNA input around 5 ng in assays was reported previously in studies [31]. We did not prefer to use very low concentrations of DNA to avoid heterozygote imbalance and allelic dropout issues [20]. Some of the obtained allelic peak height imbalances were as expected which is influenced by differences in intensity levels of the four fluorescence dyes used to label the four bases in the primer extension reaction of the SNaPshot™ chemistry. This is unavoidable unless moving away from fluorescence-based SNP-typing technologies [22].The high peak height was resolved by reducing the concentration of the respective primer. Minor shifts in the electrophoretic mobility were observed due to the incorporated base at the end of each probe and to the POP-7™ polymer. However, these shifts did not interfere with the analysis because poly-T tails increased probe spacing as consistent with other reported studies [22]. A few samples evidenced one PCR product peak with more than one colour due to pull-ups. The peak in blue produced a secondary peak in black or green; this problem is probably due to bleed-through.

We use same SNPs for multiple phenotype variants because a single SNP can affect multiple phenotypes. The proposed method elucidates the underlying associations. Their genetic underpinnings were highlighted as those SNPs were related to the same trait of interest and that is ear morphologies. It gives insights to SNP-phenotype associations and helps to find pleiotropic loci as well.

The individual’s genotype change from GG to AA in rs13427222 was 0.246 times (*p*-value = 0.022) less likely to have free lobes. The rs13427222 association with the attached ear lobe was previously reported in another study [16]. The individual genotype change from AA to GG in rs7567615 was 2.454 times (*p*-value = 0.020) more likely to cause superior helix rolling as was previously reported [16]. The possible reason might be that these genetic variations are common in the Asians, Americans and Europeans. As Europeans are considered genetically closer to Pakistanis, we hypothesized that some of the previously reported loci of ear morphology might also be associated with ear morphology in the Punjabi Pakistani population [62, 63]. Our other eighteen variants are linked to different ear phenotypes compared to the phenotypes reported by Adhikari et al. Compared to our methodology, the study reported by Adhikari et al. used a different methodology to link the ear phenotypes to the genetic variants [16]. This suggests that further validation through functional studies would be required to confirm the link of the genetic variants to the predicted phenotypes in the Punjabi population of Pakistan. The statistical non-significance of SNPs for the trait of interest suggests that those SNPs might not play a role in Pakistani population ear morphology and are non-informative. The Punjab population of Pakistan is highly conserved due to consanguinity [64] compared to the European or American admixed populations [65]. The genetic variations may not be common in Asians to account for sample size.

We oversampled young individuals in our study. In the future, however, anthropometric measurements could be taken into account for better accuracies. Analysis of full genes sequences may be important to achieve good accuracy of prediction. Closely related populations may show differences in allele frequencies affecting the significance of certain predictors and consequently affecting prediction results. Therefore, further studies on the prediction models should involve sample sets from various ethnicities in the Punjabis of Pakistanis, which may improve prediction accuracies. An additional argument is including age- and gender-dependent morphological changes in prediction modelling of appearance traits. It is still unclear how sex can affect ear phenotypes.

Based on our data, we proposed p > 0.7 as the optimal threshold which allows for increased prediction accuracy. There are fluctuations in prediction accuracies from excellent prediction, highly predictive and reasonably good predictive phenotypes. This suggests that epigenetic factors, insertion-deletion and repeated variations, pleiotropic and epistasis might be contributing to phenotypic traits. Notably, the higher values of AUC indicate the statistical model being used has higher accuracy because data was split into the training and testing sets to avoid any overfitting. Another possible reason is that as multinomial logistic regression is a useful categorical classifier and has been employed for the prediction of eye, hair and skin colour, there is a real risk of over-fitting data with small sample sizes. It is important to have enough data to avoid overfitting. Future work will be directed on a large sample size to avoid this aspect. The result obtained is a novel step towards providing Pakistan norms including data which provide the medico-legal scientist with robust classification statistics that can be easily applied when they are confronted with ear or ear prints.

## Conclusion

Ear morphologies can be predicted from biological samples using multiplex PCR assays combined with SNaPshot™ chemistry and predictive modeling, as developed in this study. A set of 21 SNPs were analysed for association with ear morphologies and revealed significant results. The study confirms independent SNP association for rs13427222 with lobe attachment prediction and rs7567615 with helix rolling in our Punjab population as previously reported in other studies of ear morphologies. However, in our study, the SNaPshot assays are shown to be good predictive for ear phenotypes in the representative Punjabi population of Pakistan. Importantly, the DNA prediction model showed higher accuracy for superior helix rolling, Darwin tubercle and lobe size prediction. Combining these SNPs into one assay for inferring hair, skin, eye colour and ear phenotypes of the Pakistani population simultaneously would be an ideal strategy for developing a phenotypic profile of multiple traits from an unknown source sample.

## Key points

1. We evaluated 21 SNPs for predictive DNA analysis of ear morphologies in the Punjab origin of the Pakistan population.
2. Two multiplex SNaPshot (Plex-1 and Plex-2) assays were developed.
3. Genotype phenotype associations and prediction models were formed.

### Supplementary Information

Below is the link to the electronic supplementary material.

Supplementary file1 (DOCX 170 KB)

## References

[1] Graham EA (2008). DNA reviews: predicting phenotype. Forensic Sci Med Pathol.

[2] Mackay TFC, Stone EA, Ayroles JF (2009). The genetics of quantitative traits: challenges and prospects. Nat Rev Genet.

[3] Frazer KA (2009). Human genetic variation and its contribution to complex traits. Nat Rev Genet.

[4] Shaffer JR (2017). Multiethnic GWAS reveals polygenic architecture of earlobe attachment. Am J Hum Genet.

[5] Verma K, Bhawana J, Vikas K (2014). Morphological variation of ear for individual identification in forensic cases: a study of an indian population. Research Journal of Forensic Sciences.

[6] Rubio O, Galera V, Alonso MC (2017). Morphological variability of the earlobe in a Spanish population sample. Homo.

[7] Kasprzak HJ (2005). Forensic otoscopy- new method of human identification. Jurisprudencija.

[8] Kasprzak J. Forensic otoscopy-new method of human identification. Jurisprudencija. 2005;(66).

[9] Nitin K. Human earprints: a review. J Biom Biostat. 2011.

[10] Guyomarc'h P, Stephan C. The validity of ear prediction guidelines used in facial approximation. J Forensic Sci. 2012;57. 10.1111/j.1556-4029.2012.02181.x 22594579

[11] Kapil V, Bhawana J, Vikas K (2014). Morphological variation of ear for individual identification in forensic cases: a study of an Indian population. Res J Forensic Sci.

[12] Zulkifli N, Yusof ZF, Rashid RA (2014). Anthropometric comparison of cross-sectional external ear between monozygotic twin. Ann Forensic Res Anal.

[13] Cox TC (2014). The genetics of auricular development and malformation: new findings in model systems driving future directions for microtia research. Eur J Med Genet.

[14] Rubio O, Galera V, Alonso MC (2019). Dependency relationships among ear characters in a Spanish sample, its forensic interest. Leg Med.

[15] Beleza-Meireles A (2014). Oculo-auriculo-vertebral spectrum: a review of the literature and genetic update. J Med Genet.

[16] Adhikari K (2015). A genome-wide association study identifies multiple loci for variation in human ear morphology. Nat Commun.

[17] Gjuvsland AB (2013). Bridging the genotype-phenotype gap: what does it take?. J Physiol.

[18] Yun L, et al. Application of six IrisPlex SNPs and comparison of two eye color prediction systems in diverse Eurasia populations. Int J Legal Med. 2014;128. 10.1007/s00414-013-0953-1 24395150

[19] Spichenok O (2010). Prediction of eye and skin color in diverse populations using seven SNPs. Forensic Sci Int Genet.

[20] Kukla-Bartoszek M (2019). DNA-based predictive models for the presence of freckles. Forensic Sci Int Genet.

[21] Draus-Barini J (2013). Bona fide colour: DNA prediction of human eye and hair colour from ancient and contemporary skeletal remains. Investig Genet.

[22] Fondevila M (2017). Forensic SNP genotyping with SNaPshot: technical considerations for the development and optimization of multiplexed SNP assays. Forensic Sci Rev.

[23] Mehta B (2017). Forensically relevant SNaPshot(®) assays for human DNA SNP analysis: a review. Int J Legal Med.

[24] Chen X, Sullivan PF (2003). Single nucleotide polymorphism genotyping: biochemistry, protocol, cost and throughput. Pharmacogenomics J.

[25] Grimes EA (2001). Sequence polymorphism in the human melanocortin 1 receptor gene as an indicator of the red hair phenotype. Forensic Sci Int.

[26] Pearson TA, Manolio TA (2008). How to interpret a genome-wide association study. JAMA.

[27] Walsh S (2011). Developmental validation of the IrisPlex system: determination of blue and brown iris colour for forensic intelligence. Forensic Sci Int Genet.

[28] Walsh S (2014). Developmental validation of the HIrisPlex system: DNA-based eye and hair colour prediction for forensic and anthropological usage. Forensic Sci Int Genet.

[29] Chaitanya L (2018). The HIrisPlex-S system for eye, hair and skin colour prediction from DNA: Introduction and forensic developmental validation. Forensic Sci Int Genet.

[30] Lettre G (2008). Identification of ten loci associated with height highlights new biological pathways in human growth. Nat Genet.

[31] Marcińska M (2015). Evaluation of DNA variants associated with androgenetic alopecia and their potential to predict male pattern baldness. PLoS ONE.

[32] Marcińska M (2015). Evaluation of DNA variants associated with androgenetic alopecia and their potential to predict male pattern baldness. PLoS ONE.

[33] Fujimoto A (2008). A replication study confirmed the EDAR gene to be a major contributor to population differentiation regarding head hair thickness in Asia. Hum Genet.

[34] Shabani M (2018). Forensic epigenetic age estimation and beyond: ethical and legal considerations. Trends Genet.

[35] Lee MK (2017). Genome-wide association study of facial morphology reveals novel associations with FREM1 and PARK2. PLoS ONE.

[36] Claes P, Hill H, Shriver MD (2014). Toward DNA-based facial composites: preliminary results and validation. Forensic Sci Int Genet.

[37] Kayser M (2015). Forensic DNA phenotyping: predicting human appearance from crime scene material for investigative purposes. Forensic Sci Int Genet.

[38] Kayser M, Schneider PM (2009). DNA-based prediction of human externally visible characteristics in forensics: motivations, scientific challenges, and ethical considerations. Forensic Sci Int Genet.

[39] Marano L, Fridman C (2019). DNA phenotyping: current application in forensic science. Res Rep Forensic Med Sci.

[40] Mateen RM, Tariq A (2019). Increasing acceptability of forensic DNA analysis in Pakistan. Egypt J Forensic Sci.

[41] Mateen RM, Tariq A, Rasool N (2018). Forensic science in Pakistan; present and future. Egypt J Forensic Sci.

[42] Matheson S (2016). DNA phenotyping: snapshot of a criminal. Cell.

[43] Mian A, Bhutta AM, Mushtaq R (1994). Genetic studies in some ethnic groups of Pakistan (Southern Punjab): colour blindness, ear lobe attachment and behavioural traits. Anthropol Anz.

[44] Rahat MA, Khan H, Hassan I, Haris M, Israr M. DNA-based eye color prediction of Pakhtun population living in District Swat KP Pakistan. Adv Life Sci. 2020.

[45] Ramzan M (2020). Spectrum of genetic variants in moderate to severe sporadic hearing loss in Pakistan. Sci Rep.

[46] Liu F (2015). Genetics of skin color variation in Europeans: genome-wide association studies with functional follow-up. Hum Genet.

[47] Sulem P (2007). Genetic determinants of hair, eye and skin pigmentation in Europeans. Nat Genet.

[48] Dembinski GM, Picard CJ (2014). Evaluation of the IrisPlex DNA-based eye color prediction assay in a United States population. Forensic Sci Int Genet.

[49] Lim S (2017). Customized multiplexing SNP panel for Korean-specific DNA phenotyping in forensic applications. Genes Genomics.

[50] Singh P, Purkait R (2009). Observations of external ear—an Indian study. Homo.

[51] Krishan K, Kanchan T, Thakur S (2019). A study of morphological variations of the human ear for its applications in personal identification. Egypt J Forensic Sci.

[52] Purkait R (2016). External ear: an analysis of its uniqueness. Egypt J Forensic Sci.

[53] Meijerman L, van der Lugt C, Maat GJ (2007). Cross-sectional anthropometric study of the external ear. J Forensic Sci.

[54] Butler JM. DNA extraction method. 2012.

[55] https://www.fishersci.co.uk/shop/products/qubit-3-0-fluorometer/15387293.

[56] Untergasser A (2007). Primer3Plus, an enhanced web interface to Primer3. Nucleic Acids Res.

[57] Hendling M, Barišić I (2019). In-silico design of DNA oligonucleotides: challenges and approaches. Comput Struct Biotechnol J.

[58] Vallone PM, Butler JM (2004). AutoDimer: a screening tool for primer-dimer and hairpin structures. Biotechniques.

[59] Shi YY, He L (2005). SHEsis, a powerful software platform for analyses of linkage disequilibrium, haplotype construction, and genetic association at polymorphism loci. Cell Res.

[60] Walsh S (2011). IrisPlex: a sensitive DNA tool for accurate prediction of blue and brown eye colour in the absence of ancestry information. Forensic Sci Int Genet.

[61] Podini D, Vallone PM (2009). SNP genotyping using multiplex single base primer extension assays. Methods Mol Biol.

[62] Quintana-Murci L (2004). Where west meets east: the complex mtDNA landscape of the southwest and Central Asian corridor. Am J Hum Genet.

[63] Rosenberg NA (2005). Clines, clusters, and the effect of study design on the inference of human population structure. PLoS Genet.

[64] Afzal M, Ali SM, Siyal HB (1994). Consanguineous marriages in Pakistan. Pak Dev Rev.

[65] Montinaro F (2015). Unravelling the hidden ancestry of American admixed populations. Nat Commun.

