# Supplementary material for: Evaluation of loci to predict ear morphology using two SNaPshot assays
Source: Forensic Sci Med Pathol. 2022 Nov 19;19(3):335–56. doi: 10.1007/s12024-022-00545-7 (PMC10518297; doi:10.1007/s12024-022-00545-7)
Supplement: Supplementary file 1 — Supplementary file1 (DOCX 170 KB) [file 12024_2022_545_MOESM1_ESM.docx]

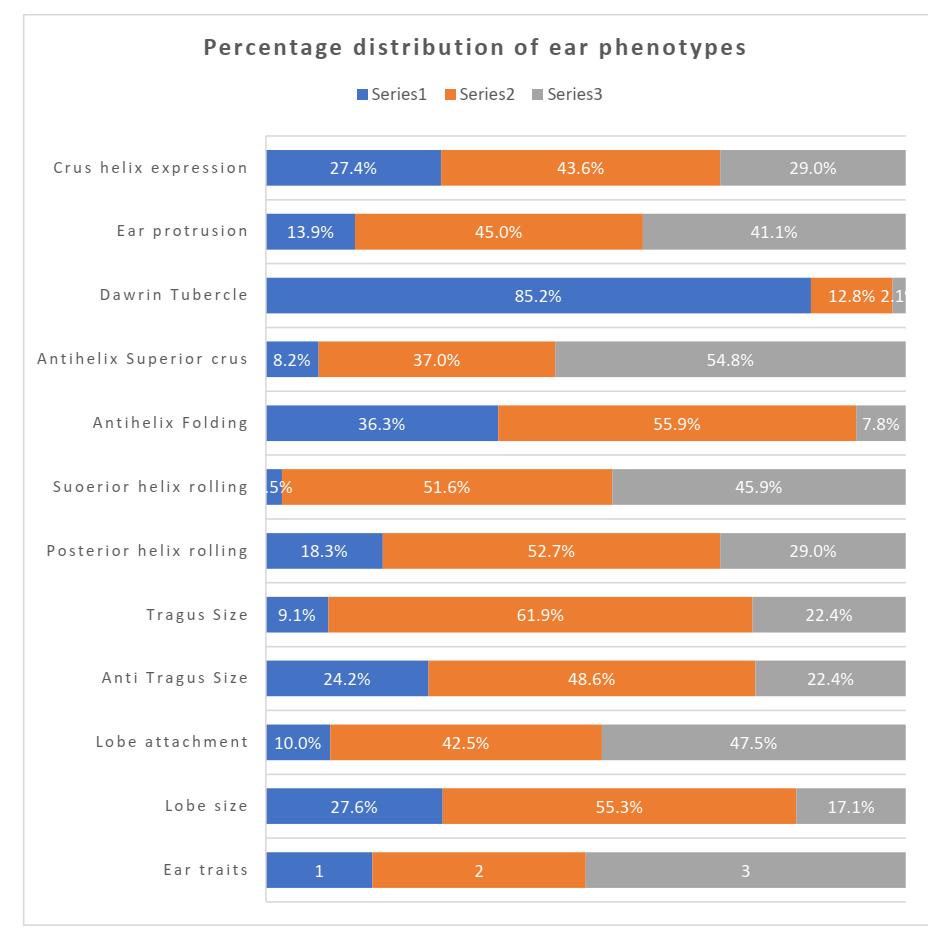


**Figure S1. Percentage distribution of phenotypes.** The colours bars indicated the differences
in percentages of phenotypic characteristics. 1-lobe size (small 27.6%, medium 55.3%, large 17.17%), 2-lobe attachment (attached lobe 10%, intermediate attachment 42.5%, free 47.5%), 3- antitragus size (absent 24.2%, average 48.6%, prominent 22.4.7%), 4- tragus size (absent 9.1%, average 61.9%, prominent 22.4.3%), 5-Posterior Helix rolling (under folded 18.3%, partial folded 52.7% and over folded 29%) , 6-superior helix rolling (under folded 5%, partial folded 51.6% and over folded 45.9%), 7-antihelix folding (under folded 36.3%, partial 55.9%, overfolded 7.8%), 8-antihelix superior crus (flat 8.2%, intermediate 37.3% and extended 54.8%), 9-darwin tubercle (absent 85.2%, degree of presence 12.8% and prominent 2.1%), 10-crus helix expression (less prominent 27.4%, prominent 43.6% and extended 29%), 11-Ear protrusion (small 13.9%, medium 45% and large 41%).

**Table S2. The results of Hardy-Weinberg equilibrium analysis.** Deviation from Hardy-Weinberg equilibrium was noted for 9 SNP (rs7567615, rs868157, rs7873690, rs1960918, rs684523, rs2378113, rs10923574, rs1619249, rs9866054) shown in as the P-values <0.05 were not consistent with HWE

| **Sr. No** | **SNP** | **No of obs. Het** | **No of Exp. Het** | **P-value** |
| --- | --- | --- | --- | --- |
| 1 | rs17023457 | 66 | 70.8 | 0.2367 |
| 2 | rs7428 | 160 | 148.9 | 0.196053 |
| 3 | rs2080401 | 139 | 138.2 | 0.921435 |
| 4 | rs3818285 | 99 | 104.6 | 0.351745 |
| 5 | rs263156 | 138 | 146.9 | 0.294399 |
| 6 | rs260674 | 73 | 75.8 | 0.522536 |
| 7 | rs10192049 | 126 | 135.3 | 0.235084 |
| 8 | rs13427222 | 94 | 98.9 | 0.395678 |
| 9 | rs1878495 | 140 | 139.5 | 0.906291 |
| 10 | rs10212419 | 122 | 129.3 | 0.328015 |
| 11 | rs13397666 | 140 | 136.1 | 0.622125 |
| 12 | rs7567615 | 35 | 101.4 | 0.0000 |
| 13 | rs868157 | 88 | 76.6 | 0.009829 |
| 14 | rs7873690 | 45 | 85.5 | 0.0000 |
| 15 | rs1960918 | 189 | 148.6 | 0.000002 |
| 16 | rs684523 | 98 | 145.9 | 0.000000 |
| 17 | rs2378113 | 109 | 130.5 | 0.004452 |
| 18 | rs10923574 | 98 | 116 | 0.006198 |
| 19 | rs1619249 | 53 | 73.3 | 0.000001 |
| 20 | rs9866054 | 53 | 93 | 0.000000 |
| 21 | rs3827760 | monomorphic | -- | -- |

**Table S3**. Prediction accuracy parameters were obtained for multinomial models with different probability thresholds applied. For each fitted model, we have extracted the maximum probability approach sensitivity, specificity PPV, NPV and predicted error at different thresholds which includes the 05, 0.55, 0.6, 0.65, 0.7, 0.8.0.85 and 0.9. For a good model sensitivity specificity, PPV and NPV are supposed to be higher while the prediction error need to be lower. *based on total samples with probabilities >threshold, MLR-multinomial logistic regression; all values in the table are expressed in %.

|  |  |  | Maximum probablity approach | p>0.5 | p>0.55 | p>0.6 | p>0.65 | p>0.7 | p>0.75 | p>0.8 | p>0.85 | p>0.9 |
| --- | --- | --- | --- | --- | --- | --- | --- | --- | --- | --- | --- | --- |
| MLR | Small Lobe size | Sensitivity | 56.8 | 59.4 | 60.6 | 62.1 | 65.3 | 71.3 | 81.1 | 93.9 | 100 | 100 |
|  |  | Specificity | 81.5 | 82.5 | 85.6 | 88.1 | 89.7 | 90.8 | 93.7 | 96.4 | 97.9 | 100 |
|  |  | PPV | 62.3 | 64.6 | 68 | 70.6 | 73.2 | 78.8 | 87.5 | 90 | 93.9 | 100 |
|  |  | NPV | 77.8 | 79 | 81.3 | 83.3 | 85.6 | 86.6 | 90.2 | 98 | 99.9 | 100 |
|  |  | Prediction error* | 29.3 | 27.8 | 24.8 | 22.4 | 19.9 | 17.7 | 12.8 | 6.6 | 3.3 | 0 |
|  | Medium lobe size | Sensitivity | 76.9 | 78.1 | 81.6 | 84.8 | 86.5 | 86.8 | 86.6 | 89.2 | 95.5 | 100 |
|  |  | Specificity | 50.3 | 51.4 | 51.3 | 52.9 | 55.4 | 64.6 | 78.2 | 84.2 | 93.3 | 100 |
|  |  | PPV | 63 | 63.8 | 65.8 | 68.4 | 71 | 74.1 | 80.3 | 83 | 91 | 100 |
|  |  | NPV | 66.3 | 68 | 70.9 | 74 | 76.2 | 80.8 | 85 | 89.7 | 96.9 | 100 |
|  |  | Prediction error* | 37.9 | 36.8 | 34.6 | 31.9 | 29.4 | 25.3 | 19.6 | 15.4 | 7.8 | 0 |
|  | Large lobe size | Sensitivity | 18.1 | 18 | 19.3 | 22.5 | 24.7 | 42.2 | 67.7 | 74.2 | 86.7 | 100 |
|  |  | Specificity | 99.1 | 99.5 | 99.7 | 99.8 | 99.5 | 99 | 97.2 | 98.4 | 99.7 | 100 |
|  |  | PPV | 56.7 | 62.3 | 68.4 | 72.8 | 71.7 | 74.9 | 77.4 | 88.5 | 100 | 100 |
|  |  | NPV | 90.4 | 90.4 | 90.1 | 90.3 | 90.3 | 92.6 | 95.6 | 92.5 | 95.1 | 100 |
|  |  | Prediction error* | 13 | 12.7 | 12.7 | 12.5 | 12.6 | 10.7 | 8.9 | 9.9 | 5.6 | 0 |
|  |  | Inconclusive | 0 | 15.3 | 33.9 | 54.1 | 68.8 | 81.3 | 89.4 | 93.5 | 95.5 | 96.3 |

|  |  |  | The highest probability approach | p>0.5 | p>0.55 | p>0.6 | p>0.65 | p>0.7 | p>0.75 | p>0.8 | p>0.85 | p>0.9 |
| --- | --- | --- | --- | --- | --- | --- | --- | --- | --- | --- | --- | --- |
| MLR | Attached ear lobe | Sensitivity | 24.1 | 24 | 25.3 | 28.5 | 30.7 | 48.2 | 73.7 | 80.2 | 92.7 | 100 |
|  |  | Specificity | 99.1 | 99.5 | 99.7 | 99.8 | 99.5 | 99 | 97.2 | 98.4 | 100 | 100 |
|  |  | PPV | 56.7 | 62.3 | 68.4 | 72.8 | 71.7 | 74.9 | 77.4 | 88.5 | 100 | 100 |
|  |  | NPV | 90.4 | 90.4 | 90.1 | 90.3 | 90.3 | 92.6 | 95.6 | 92.5 | 95.1 | 100 |
|  |  | Prediction error* | 13 | 12.7 | 12.7 | 12.5 | 12.6 | 10.7 | 8.9 | 9.9 | 5.6 | 0 |
|  | Average  atttached | Sensitivity | 56.8 | 59.4 | 60.6 | 62.1 | 65.3 | 71.3 | 81.1 | 93.9 | 99.5 | 100 |
|  |  | Specificity | 81.5 | 82.5 | 85.6 | 88.1 | 89.7 | 90.8 | 93.7 | 96.4 | 97.9 | 100 |
|  |  | PPV | 62.3 | 64.6 | 68 | 70.6 | 73.2 | 78.8 | 87.5 | 90 | 93.9 | 100 |
|  |  | NPV | 77.8 | 79 | 81.3 | 83.3 | 85.6 | 86.6 | 90.2 | 98 | 99.6 | 100 |
|  |  | Prediction error* | 29.3 | 27.8 | 24.8 | 22.4 | 19.9 | 17.7 | 12.8 | 6.6 | 3.3 | 0 |
|  | Free ear lobe | Sensitivity | 76.9 | 78.1 | 81.6 | 84.8 | 86.5 | 86.8 | 86.6 | 89.2 | 95.5 | 100 |
|  |  | Specificity | 50.3 | 51.4 | 51.3 | 52.9 | 55.4 | 64.6 | 78.2 | 84.2 | 93.3 | 100 |
|  |  | PPV | 63 | 63.8 | 65.8 | 68.4 | 71 | 74.1 | 80.3 | 83 | 91 | 100 |
|  |  | NPV | 66.3 | 68 | 70.9 | 74 | 76.2 | 80.8 | 85 | 89.7 | 96.9 | 100 |
|  |  | Prediction error* | 37.9 | 36.8 | 34.6 | 31.9 | 29.4 | 25.3 | 19.6 | 15.4 | 7.8 | 0 |
|  |  | Inconclusive | 0 | 16.6 | 35.2 | 55.4 | 70.1 | 82.6 | 90.7 | 94.8 | 96.8 | 97.6 |

|  |  |  | The highest probability approach | p>0.5 | p>0.55 | p>0.6 | p>0.65 | p>0.7 | p>0.75 | p>0.8 | p>0.85 | p>0.9 |
| --- | --- | --- | --- | --- | --- | --- | --- | --- | --- | --- | --- | --- |
| MLR | Absent antitragus | Sensitivity | 78.2 | 79.4 | 82.9 | 86.1 | 87.8 | 88.1 | 87.9 | 90.5 | 96.8 | 100 |
|  |  | Specificity | 51.6 | 52.7 | 52.6 | 54.2 | 56.7 | 65.9 | 79.5 | 85.5 | 94.6 | 100 |
|  |  | PPV | 64.3 | 65.1 | 67.1 | 69.7 | 72.3 | 75.4 | 81.6 | 84.3 | 92.3 | 100 |
|  |  | NPV | 67.6 | 69.3 | 72.2 | 75.3 | 77.5 | 82.1 | 86.3 | 91 | 98.2 | 100 |
|  |  | Prediction error* | 39.2 | 38.1 | 35.9 | 33.2 | 30.7 | 26.6 | 20.9 | 16.7 | 9.1 | 0 |
|  | Average antitragus | Sensitivity | 19.4 | 19.3 | 20.6 | 23.8 | 26 | 43.5 | 69 | 75.5 | 88 | 100 |
|  |  | Specificity | 95.8 | 96.2 | 96.4 | 96.5 | 96.2 | 95.7 | 93.9 | 95.1 | 97.7 | 100 |
|  |  | PPV | 58 | 63.6 | 69.7 | 74.1 | 73 | 76.2 | 78.7 | 89.8 | 100 | 100 |
|  |  | NPV | 91.7 | 91.7 | 91.4 | 91.6 | 91.6 | 93.9 | 96.9 | 93.8 | 96.4 | 100 |
|  |  | Prediction error* | 14.3 | 14 | 14 | 13.8 | 13.9 | 12 | 10.2 | 11.2 | 6.9 | 0 |
|  | prominent | Sensitivity | 58.1 | 60.7 | 61.9 | 63.4 | 66.6 | 72.6 | 82.4 | 95.2 | 99.3 | 100 |
|  |  | Specificity | 82.8 | 83.8 | 86.9 | 89.4 | 91 | 92.1 | 95 | 97.7 | 99.2 | 100 |
|  |  | PPV | 63.6 | 65.9 | 69.3 | 71.9 | 74.5 | 80.1 | 88.8 | 91.3 | 95.2 | 100 |
|  |  | NPV | 79.1 | 80.3 | 82.6 | 84.6 | 86.9 | 87.9 | 91.5 | 99.3 | 100 | 100 |
|  |  | Prediction error* | 30.6 | 29.1 | 26.1 | 23.7 | 21.2 | 19 | 14.1 | 7.9 | 4.6 | 0 |
|  |  | Inconclusive | 0 | 18.3 | 36.9 | 57.1 | 71.8 | 84.3 | 92.4 | 96.5 | 98.5 | 99.3 |

|  |  |  | The highest probability approach | p>0.5 | p>0.55 | p>0.6 | p>0.65 | p>0.7 | p>0.75 | p>0.8 | p>0.85 | p>0.9 |
| --- | --- | --- | --- | --- | --- | --- | --- | --- | --- | --- | --- | --- |
| MLR | Absent tragus | Sensitivity | 49.2 | 50.7 | 51.05 | 56.45 | 56.95 | 68 | 78.7 | 83.85 | 90.8 | 100 |
|  |  | Specificity | 73.95 | 74.65 | 75.35 | 74.7 | 76.85 | 76.8 | 84.9 | 90.1 | 92.8 | 100 |
|  |  | PPV | 59 | 62.45 | 65.7 | 69.65 | 70 | 73.25 | 76 | 86.25 | 90.5 | 100 |
|  |  | NPV | 76.95 | 78.7 | 78.5 | 81.6 | 81.65 | 85 | 88.9 | 89.1 | 93.4 | 100 |
|  |  | Prediction error* | 25.45 | 24.3 | 24.15 | 22.05 | 21.35 | 18.65 | 15.45 | 12.55 | 9.6 | 0 |
|  | Average tragus | Sensitivity | 37 | 38.2 | 38.75 | 40.9 | 44.3 | 47.7 | 69.7 | 84.45 | 86.2 | 100 |
|  |  | Specificity | 89.65 | 90.45 | 92.1 | 93.45 | 94.2 | 94.55 | 95.7 | 95.55 | 98.95 | 100 |
|  |  | PPV | 56.8 | 62.3 | 65.25 | 71.4 | 72.3 | 74.4 | 82.65 | 82.5 | 96.95 | 100 |
|  |  | NPV | 83.7 | 84.1 | 85.4 | 86 | 87.75 | 87.6 | 92.35 | 96.35 | 94.6 | 100 |
|  |  | Prediction error* | 20.65 | 19.85 | 18.1 | 17.2 | 15.4 | 15 | 9.9 | 7.65 | 6.2 | 0 |
|  | Prominent tragus | Sensitivity | 65.7 | 67.6 | 70.6 | 72.2 | 74.5 | 76.45 | 80.5 | 87.45 | 97.75 | 100 |
|  |  | Specificity | 64.85 | 66.5 | 66.35 | 70.35 | 70.85 | 77.8 | 83.35 | 90.55 | 93.55 | 100 |
|  |  | PPV | 61.3 | 63.4 | 64.95 | 69.15 | 70.15 | 74.55 | 80.45 | 87.65 | 88.35 | 100 |
|  |  | NPV | 71.1 | 72.8 | 74.65 | 78.05 | 79.3 | 83.8 | 84.7 | 91.8 | 98.45 | 100 |
|  |  | Prediction error* | 33.7 | 31.95 | 30.5 | 26.55 | 25.45 | 20.8 | 18.3 | 10.95 | 6.2 | 0 |
|  |  | Inconclusive | 0 | 14.45 | 29.7 | 52.45 | 67.4 | 80.75 | 90.15 | 95 | 97.25 | 98.45 |

|  |  |  | The highest probability approach | p>0.5 | p>0.55 | p>0.6 | p>0.65 | p>0.7 | p>0.75 | p>0.8 | p>0.85 | p>0.9 |
| --- | --- | --- | --- | --- | --- | --- | --- | --- | --- | --- | --- | --- |
| MLR | Underfolded posterior helix | Sensitivity | 76.25 | 78.4 | 79.85 | 85.25 | 85.5 | 87.95 | 85.8 | 89 | 92.85 | 100 |
|  |  | Specificity | 50.2 | 51.25 | 51.8 | 51.9 | 55.45 | 60.25 | 76.05 | 83.65 | 89.6 | 100 |
|  |  | PPV | 62.8 | 63.85 | 65.05 | 68.1 | 70.3 | 73.5 | 78.1 | 84.15 | 86.15 | 100 |
|  |  | NPV | 65.55 | 68.15 | 69.55 | 74.1 | 75.25 | 79.75 | 84.25 | 88.35 | 94.95 | 100 |
|  |  | Prediction error* | 38.55 | 37 | 35.75 | 32.4 | 30.4 | 26.6 | 21.45 | 15.95 | 11.35 | 0 |
|  | Partial folded posterior helix rolling | Sensitivity | 18.3 | 18.15 | 18.75 | 21.75 | 24.65 | 33.8 | 63.65 | 75.25 | 79.7 | 100 |
|  |  | Specificity | 96.8 | 97.3 | 97.5 | 97.65 | 97.45 | 97 | 95.8 | 94.9 | 98.85 | 100 |
|  |  | PPV | 54.65 | 61.8 | 66.1 | 73.15 | 72.2 | 73.1 | 78.25 | 82.4 | 100 | 100 |
|  |  | NPV | 90.65 | 90.45 | 90.45 | 90.15 | 90.75 | 91.25 | 95.7 | 94.25 | 92.3 | 100 |
|  |  | Prediction error* | 13.15 | 12.95 | 12.7 | 12.9 | 12.4 | 12.15 | 8.6 | 9.95 | 8 | 0 |
|  | Over folded posterior helix rolling | Sensitivity | 56.3 | 58.9 | 60.75 | 61.5 | 64.55 | 69.35 | 78.4 | 90.45 | 100 | 100 |
|  |  | Specificity | 81.1 | 82.7 | 84.15 | 88.6 | 88.65 | 91.55 | 91.75 | 97.3 | 96.5 | 100 |
|  |  | PPV | 61.6 | 64.45 | 66.7 | 70.9 | 71.9 | 77.55 | 84.7 | 91.8 | 90.45 | 100 |
|  |  | NPV | 77.5 | 78.95 | 80.5 | 83.35 | 84.65 | 87.35 | 87.95 | 96.6 | 100 | 100 |
|  |  | Prediction error* | 30.05 | 28.1 | 26.25 | 22.45 | 21.35 | 17.65 | 15.55 | 7.2 | 4.6 | 0 |
|  |  | Inconclusive | 0 | 15.3 | 30.55 | 53.3 | 68.25 | 81.6 | 91 | 95.85 | 98.1 | 99.3 |

|  |  |  | The highest probability approach | p>0.5 | p>0.55 | p>0.6 | p>0.65 | p>0.7 | p>0.75 | p>0.8 | p>0.85 | p>0.9 |
| --- | --- | --- | --- | --- | --- | --- | --- | --- | --- | --- | --- | --- |
| MLR | Underfolded superior helix rolling | Sensitivity | 61.75 | 64.05 | 63.925 | 70.425 | 70.075 | 77.9 | 81.2 | 85.675 | 89.85 | 100 |
|  |  | Specificity | 61.375 | 62.225 | 63.175 | 62.15 | 65.525 | 65.7 | 78.75 | 85.95 | 88.7 | 100 |
|  |  | PPV | 60.15 | 62.525 | 64.35 | 68.075 | 69.15 | 72.425 | 75.3 | 85.125 | 85.25 | 100 |
|  |  | NPV | 70.225 | 72.85 | 72.7 | 77.25 | 77.325 | 81.2 | 85.55 | 87.4 | 92.55 | 100 |
|  |  | Prediction error* | 31.675 | 30.1 | 29.875 | 26.825 | 25.725 | 22.625 | 18.725 | 13.875 | 11.6 | 0 |
|  | Partialsuperior helix rolling | Sensitivity | 27.1 | 27.6 | 27.825 | 30.3 | 33.8 | 35.9 | 64 | 79.725 | 78.8 | 100 |
|  |  | Specificity | 93.725 | 94.425 | 95.35 | 96.125 | 96.45 | 96.425 | 96.7 | 95.125 | 99.475 | 100 |
|  |  | PPV | 54.05 | 61.15 | 63.875 | 71.8 | 71.85 | 72.2 | 80.225 | 78.75 | 98.475 | 100 |
|  |  | NPV | 86.65 | 86.65 | 87.45 | 87.35 | 88.825 | 88.1 | 93.425 | 95.525 | 91.4 | 100 |
|  |  | Prediction error* | 16.325 | 15.875 | 14.75 | 14.6 | 13.15 | 13.65 | 8.45 | 8.175 | 7.65 | 0 |
|  | Over folded superior helix rolling | Sensitivity | 60.1 | 62.35 | 65.1 | 65.9 | 68.5 | 71.275 | 77.45 | 86.575 | 98.875 | 100 |
|  |  | Specificity | 72.125 | 74.05 | 73.875 | 79.075 | 78.575 | 84.4 | 85.925 | 93.725 | 93.675 | 100 |
|  |  | PPV | 60.45 | 63.2 | 64.525 | 69.525 | 69.725 | 74.775 | 80.525 | 89.975 | 87.025 | 100 |
|  |  | NPV | 73.5 | 75.2 | 76.525 | 80.075 | 80.85 | 85.3 | 84.55 | 92.85 | 99.225 | 100 |
|  |  | Prediction error* | 31.6 | 29.525 | 28.45 | 23.875 | 23.475 | 18.55 | 17.65 | 8.725 | 5.4 | 0 |
|  |  | Inconclusive | 0 | 13.375 | 26.95 | 50.975 | 66.05 | 79.825 | 89.875 | 95.1 | 97.475 | 98.875 |

|  |  |  | The highest probability approach | p>0.5 | p>0.55 | p>0.6 | p>0.65 | p>0.7 | p>0.75 | p>0.8 | p>0.85 | p>0.9 |
| --- | --- | --- | --- | --- | --- | --- | --- | --- | --- | --- | --- | --- |
| MLR | Underfolded antihelix folding | Sensitivity | 68.025 | 70.725 | 70.3625 | 77.4125 | 76.6375 | 82.85 | 82.45 | 86.5875 | 89.375 | 100 |
|  |  | Specificity | 55.0875 | 56.0125 | 57.0875 | 55.875 | 59.8625 | 60.15 | 75.675 | 83.875 | 86.65 | 100 |
|  |  | PPV | 60.725 | 62.5625 | 63.675 | 67.2875 | 68.725 | 72.0125 | 74.95 | 84.5625 | 82.625 | 100 |
|  |  | NPV | 66.8625 | 69.925 | 69.8 | 75.075 | 75.1625 | 79.3 | 83.875 | 86.55 | 92.125 | 100 |
|  |  | Prediction error* | 34.7875 | 33 | 32.7375 | 29.2125 | 27.9125 | 24.6125 | 20.3625 | 14.5375 | 12.6 | 0 |
|  | Partial folded antihelix folding | Sensitivity | 22.15 | 22.3 | 22.3625 | 25 | 28.55 | 30 | 61.15 | 77.3625 | 75.1 | 100 |
|  |  | Specificity | 95.7625 | 96.4125 | 96.975 | 97.4625 | 97.575 | 97.3625 | 97.2 | 94.9125 | 99.7375 | 100 |
|  |  | PPV | 52.675 | 60.575 | 63.1875 | 72 | 71.625 | 71.1 | 79.0125 | 76.875 | 99.2375 | 100 |
|  |  | NPV | 88.125 | 87.925 | 88.475 | 88.025 | 89.3625 | 88.35 | 93.9625 | 95.1125 | 89.8 | 100 |
|  |  | Prediction error* | 14.1625 | 13.8875 | 13.075 | 13.3 | 12.025 | 12.975 | 7.725 | 8.4375 | 8.375 | 0 |
|  | Overfolded Antihelix folding | Sensitivity | 57.3 | 59.725 | 62.35 | 62.75 | 65.5 | 68.6875 | 75.925 | 86.1375 | 99.4375 | 100 |
|  |  | Specificity | 75.7625 | 77.825 | 77.6375 | 83.4375 | 82.4375 | 87.7 | 87.2125 | 95.3125 | 93.7375 | 100 |
|  |  | PPV | 60.025 | 63.1 | 64.3125 | 69.7125 | 69.5125 | 74.8875 | 80.5625 | 91.1375 | 86.3625 | 100 |
|  |  | NPV | 74.7 | 76.4 | 77.4625 | 81.0875 | 81.625 | 86.05 | 84.475 | 93.375 | 99.6125 | 100 |
|  |  | Prediction error* | 30.55 | 28.3125 | 27.425 | 22.5375 | 22.4875 | 17.425 | 17.325 | 7.6125 | 5 | 0 |
|  |  | Inconclusive | 0 | 12.8375 | 25.575 | 50.2375 | 65.375 | 79.3625 | 89.7375 | 95.15 | 97.5875 | 99.0875 |

|  |  |  | The highest probability approach | p>0.5 | p>0.55 | p>0.6 | p>0.65 | p>0.7 | p>0.75 | p>0.8 | p>0.85 | p>0.9 |
| --- | --- | --- | --- | --- | --- | --- | --- | --- | --- | --- | --- | --- |
| MLR | Flat antihelix superior crus | Sensitivity | 53.38 | 55.15 | 55.34 | 61.11 | 61.33 | 71.3 | 79.53 | 84.46 | 90.48 | 100 |
|  |  | Specificity | 69.76 | 70.51 | 71.29 | 70.52 | 73.08 | 73.1 | 82.85 | 88.72 | 91.43 | 100 |
|  |  | PPV | 59.38 | 62.48 | 65.25 | 69.13 | 69.72 | 72.98 | 75.77 | 85.88 | 88.75 | 100 |
|  |  | NPV | 74.71 | 76.75 | 76.57 | 80.15 | 80.21 | 83.73 | 87.78 | 88.53 | 93.12 | 100 |
|  |  | Prediction error* | 27.53 | 26.23 | 26.06 | 23.64 | 22.81 | 19.98 | 16.54 | 12.99 | 10.27 | 0 |
|  | Averagesantihelix superior crus | Sensitivity | 33.7 | 34.67 | 35.11 | 37.37 | 40.8 | 43.77 | 67.8 | 82.88 | 83.73 | 100 |
|  |  | Specificity | 91.01 | 91.78 | 93.18 | 94.34 | 94.95 | 95.18 | 96.03 | 95.41 | 99.13 | 100 |
|  |  | PPV | 55.88 | 61.92 | 64.79 | 71.53 | 72.15 | 73.67 | 81.84 | 81.25 | 97.46 | 100 |
|  |  | NPV | 84.68 | 84.95 | 86.08 | 86.45 | 88.11 | 87.77 | 92.71 | 96.08 | 93.53 | 100 |
|  |  | Prediction error* | 19.21 | 18.53 | 16.98 | 16.33 | 14.65 | 14.55 | 9.42 | 7.83 | 6.68 | 0 |
|  | Extended Antihelix superior crus | Sensitivity | 63.83 | 65.85 | 68.77 | 70.1 | 72.5 | 74.73 | 79.48 | 87.16 | 98.13 | 100 |
|  |  | Specificity | 67.28 | 69.02 | 68.86 | 73.26 | 73.43 | 80 | 84.21 | 91.61 | 93.59 | 100 |
|  |  | PPV | 61.02 | 63.33 | 64.81 | 69.28 | 70.01 | 74.63 | 80.48 | 88.43 | 87.91 | 100 |
|  |  | NPV | 71.9 | 73.6 | 75.28 | 78.73 | 79.82 | 84.3 | 84.65 | 92.15 | 98.71 | 100 |
|  |  | Prediction error* | 33 | 31.14 | 29.82 | 25.66 | 24.79 | 20.05 | 18.08 | 10.21 | 5.93 | 0 |
|  |  | Inconclusive | 0 | 14.09 | 28.78 | 51.96 | 66.95 | 80.44 | 90.06 | 95.03 | 97.33 | 98.59 |

|  |  |  | The highest probability approach | p>0.5 | p>0.55 | p>0.6 | p>0.65 | p>0.7 | p>0.75 | p>0.8 | p>0.85 | p>0.9 |
| --- | --- | --- | --- | --- | --- | --- | --- | --- | --- | --- | --- | --- |
| MLR | Absent Darwin tubercle | Sensitivity | 61.75 | 64.05 | 63.93 | 70.43 | 70.08 | 77.9 | 81.2 | 85.68 | 89.85 | 100 |
|  |  | Specificity | 61.38 | 62.23 | 63.18 | 62.15 | 65.53 | 65.7 | 78.75 | 85.95 | 88.7 | 100 |
|  |  | PPV | 60.15 | 62.53 | 64.35 | 68.08 | 69.15 | 72.43 | 75.3 | 85.13 | 85.25 | 100 |
|  |  | NPV | 70.23 | 72.85 | 72.7 | 77.25 | 77.33 | 81.2 | 85.55 | 87.4 | 92.55 | 100 |
|  |  | Prediction error* | 31.68 | 30.1 | 29.88 | 26.83 | 25.73 | 22.63 | 18.73 | 13.88 | 11.6 | 0 |
|  | Average Darwin Tubercle | Sensitivity | 27.1 | 27.6 | 27.83 | 30.3 | 33.8 | 35.9 | 64 | 79.73 | 78.8 | 100 |
|  |  | Specificity | 93.73 | 94.43 | 95.35 | 96.13 | 96.45 | 96.43 | 96.7 | 95.13 | 99.48 | 100 |
|  |  | PPV | 54.05 | 61.15 | 63.88 | 71.8 | 71.85 | 72.2 | 80.23 | 78.75 | 98.48 | 100 |
|  |  | NPV | 86.65 | 86.65 | 87.45 | 87.35 | 88.83 | 88.1 | 93.43 | 95.53 | 91.4 | 100 |
|  |  | Prediction error* | 16.33 | 15.88 | 14.75 | 14.6 | 13.15 | 13.65 | 8.45 | 8.18 | 7.65 | 0 |
|  | Prominent Darwin Tubercle | Sensitivity | 60.1 | 62.35 | 65.1 | 65.9 | 68.5 | 71.28 | 77.45 | 86.58 | 98.88 | 100 |
|  |  | Specificity | 72.13 | 74.05 | 73.88 | 79.08 | 78.58 | 84.4 | 85.93 | 93.73 | 93.68 | 100 |
|  |  | PPV | 60.45 | 63.2 | 64.53 | 69.53 | 69.73 | 74.78 | 80.53 | 89.98 | 87.03 | 100 |
|  |  | NPV | 73.5 | 75.2 | 76.53 | 80.08 | 80.85 | 85.3 | 84.55 | 92.85 | 99.23 | 100 |
|  |  | Prediction error* | 31.6 | 29.53 | 28.45 | 23.88 | 23.48 | 18.55 | 17.65 | 8.73 | 5.4 | 0 |
|  |  | Inconclusive | 0 | 13.38 | 26.95 | 50.98 | 66.05 | 79.83 | 89.88 | 95.1 | 97.48 | 98.88 |

|  |  |  | The highest probability approach | p>0.5 | p>0.55 | p>0.6 | p>0.65 | p>0.7 | p>0.75 | p>0.8 | p>0.85 | p>0.9 |
| --- | --- | --- | --- | --- | --- | --- | --- | --- | --- | --- | --- | --- |
| MLR | Small Crus Helix expression | Sensitivity | 60.56 | 61.71 | 63.76 | 67.78 | 69.09 | 75.58 | 82.68 | 86.72 | 94.04 | 100 |
|  |  | Specificity | 65.92 | 66.78 | 67.02 | 67.59 | 69.61 | 74.13 | 83.74 | 88.84 | 94.73 | 100 |
|  |  | PPV | 61.36 | 63.76 | 66.74 | 70.07 | 71.36 | 74.53 | 78.98 | 85.56 | 92.71 | 100 |
|  |  | NPV | 73.96 | 75.46 | 76.8 | 79.54 | 80.66 | 84.5 | 88.44 | 90.48 | 96.01 | 100 |
|  |  | Prediction error* | 30.77 | 29.75 | 28.59 | 26.43 | 24.93 | 21.63 | 17.36 | 14.29 | 8.85 | 0 |
|  | Medium crus helix expression | Sensitivity | 30.68 | 31.4 | 32.41 | 35 | 37.78 | 48.55 | 70.78 | 81.16 | 88.95 | 100 |
|  |  | Specificity | 91.71 | 92.33 | 93.44 | 94.31 | 94.64 | 94.66 | 94.55 | 95.43 | 98.19 | 100 |
|  |  | PPV | 58.09 | 63.24 | 67.82 | 72.65 | 72.76 | 75.85 | 81.28 | 87.09 | 99.24 | 100 |
|  |  | NPV | 86.96 | 87.26 | 87.89 | 88.46 | 89.41 | 90.63 | 94.36 | 95.28 | 96.3 | 100 |
|  |  | Prediction error* | 18.56 | 17.92 | 16.89 | 16.15 | 15.21 | 13.84 | 10.41 | 9.29 | 6.19 | 0 |
|  | Large crus helix expression | Sensitivity | 63.3 | 65.46 | 67.63 | 69.38 | 72.05 | 75.82 | 82.21 | 91.54 | 99.74 | 100 |
|  |  | Specificity | 72.01 | 73.26 | 74.74 | 77.69 | 78.99 | 83.3 | 88.53 | 93.33 | 96.34 | 100 |
|  |  | PPV | 62.66 | 64.7 | 67.23 | 70.43 | 72.43 | 77.27 | 84.61 | 88.89 | 92.11 | 100 |
|  |  | NPV | 74.5 | 75.95 | 78.16 | 80.82 | 82.71 | 85.48 | 88.14 | 95.29 | 100 | 100 |
|  |  | Prediction error* | 32.68 | 31.13 | 28.81 | 25.79 | 23.82 | 20.46 | 16.36 | 9.98 | 5.6 | 0 |
|  |  | Inconclusive | 0 | 16.64 | 33.99 | 55.14 | 69.94 | 82.76 | 91.34 | 95.73 | 97.82 | 98.77 |

|  |  |  | The highest probability approach | p>0.5 | p>0.55 | p>0.6 | p>0.65 | p>0.7 | p>0.75 | p>0.8 | p>0.85 | p>0.9 |
| --- | --- | --- | --- | --- | --- | --- | --- | --- | --- | --- | --- | --- |
| MLR | flat | Sensitivity | 48.82 | 49.6 | 51.18 | 55.7 | 57.38 | 69.16 | 80.72 | 85.35 | 93.07 | 100 |
|  |  | Specificity | 67.96 | 68.84 | 69.2 | 69.53 | 71.77 | 75.4 | 84.74 | 89.76 | 94.63 | 100 |
|  |  | PPV | 60.3 | 63.3 | 66.6 | 70.16 | 71.04 | 74.23 | 78.06 | 85.96 | 92.63 | 100 |
|  |  | NPV | 75.43 | 76.99 | 77.91 | 80.69 | 81.49 | 85.15 | 89.04 | 90.27 | 95.26 | 100 |
|  |  | Prediction error* | 25.27 | 24.42 | 23.88 | 22.33 | 21.51 | 18.6 | 15.16 | 13.19 | 8.39 | 0 |
|  | Medium Protrusion | Sensitivity | 31.02 | 31.63 | 32.63 | 35.51 | 38.57 | 48.11 | 71.01 | 82.68 | 88.81 | 100 |
|  |  | Specificity | 90.11 | 90.83 | 92.32 | 93.49 | 94.06 | 94.27 | 94.76 | 95.54 | 98.36 | 100 |
|  |  | PPV | 58.02 | 63.1 | 67.15 | 72.15 | 72.68 | 75.68 | 82.05 | 86.02 | 98.16 | 100 |
|  |  | NPV | 85.19 | 85.62 | 86.62 | 87.35 | 88.64 | 89.48 | 93.47 | 95.76 | 96.19 | 100 |
|  |  | Prediction error* | 18.98 | 18.35 | 17.24 | 16.53 | 15.38 | 14.26 | 10.33 | 8.45 | 5.58 | 0 |
|  | Large Protruison | Sensitivity | 64.51 | 66.61 | 69.02 | 70.76 | 73.35 | 76.58 | 82.06 | 90.25 | 98.85 | 100 |
|  |  | Specificity | 66.97 | 68.32 | 69.06 | 72.05 | 73.44 | 79.48 | 86.1 | 91.7 | 95.35 | 100 |
|  |  | PPV | 62.33 | 64.29 | 66.51 | 69.93 | 71.71 | 76.28 | 83.12 | 88.02 | 91.01 | 100 |
|  |  | NPV | 72.78 | 74.33 | 76.53 | 79.44 | 81.2 | 84.62 | 86.96 | 93.87 | 99.45 | 100 |
|  |  | Prediction error* | 33.22 | 31.62 | 29.51 | 26.23 | 24.46 | 20.74 | 16.96 | 10.2 | 5.79 | 0 |
|  |  | Inconclusive | 0 | 15.96 | 32.71 | 54.43 | 69.27 | 82.22 | 90.99 | 95.46 | 97.59 | 98.59 |

**Table 2: Complete Explanation**

As demonstrated in (Table 2), subjects were 0.100 times less likely (p-value<0.001) to have small lobe size as compared to large lobe size, 0.214 times less likely (p-value=0.016) to have intermediate lobe size compared to large lobe size. The highest statistical significance was obtained for the seven SNPs including rs17023457, rs13397666, rs1960918, rs1619249, rs9866054, rs13427222 and rs1878495, explaining the variation in lobe size. The genotype changed from CC to TT in rs17023457, 3.049 times (p- value=0.045) more likely to have large lobe size. The genotype changed from GG to AG in rs13397666 making it 0.454 times (p-value=0.043), from CC to CT in rs1960918 making it 0.466 time (p-value=0.042), from CC to CT in rs1619249 making it 0.180 times (p-value= 0.031), from AA to AG in rs9866054 making it 0.376 times (p-value=0.041), from GG to AG in rs13427222 making it 0.150 times (p-value=0.001), from GG to AA in rs13427222 making it 0.221 times (p-value=0.009) and from AA to CC in rs1878495 making it 0.457 times (p-value=0.044), less likely to have large lobe size.

The subjects were 0.712 times less likely (p-value=0.861) to have attached ear lobes as compared to free ear lobes, and 5.318 times more likely (p-value=0.049) to have intermediate ear lobes when compared with free ear lobes. The highest statistical significance was obtained for the four SNPs (rs7873690, rs1960918, rs1619249, rs13427222) shown an association with the attached ear lobe. The genotype changes from TT to CT in rs7873690 making it 2.654 times (p-value= 0.045), from CC to CT in rs1619249 making it 1.91 times (p-value=0.002), from CC to TT in rs1619249 making it 4.376 times, more likely to get free ear lobes. Genotype change from CC to CT in rs1960918 making it 0.493 times (p-value= 0.042), from GG to AG in rs13427222 making it 0.249 times (p-value= 0.024), from GG to AA in rs13427222 making it 0.246 times (p-value=0.022) less likely to have free lobes.

The subjects were more likely to have absent antitragus 1.708 times (p- value= 0.760) compared to prominent antitragus and 7.59 times (p-value= 0.049) more likely to have average anti-tragus when compared to prominent. The highest statistical significance was obtained for the three SNPs (rs868157, rs7873690, rs13427222), explaining the variation in antitragus size. The genotype changed from GG to TT in rs868157 which is 5.159 times (p-value=0.049), from GG to AA in rs13427222 which is 2.76 times (p-value= 0.045) more likely to get prominent antitragus. The genotype change from TT to CT in rs7873690 makes it 0.328 times (p-value=0.015) less likely to get prominent antitragus.

Subjects were 2.540 times (p-value=0.605) more likely to get absent tragus as compare with prominent tragus and 6.083 times (p-value=0.024) more likely to get average tragus when compared with prominent. The highest statistical significance was obtained for seven SNPs (rs17023457, rs868157, rs7428, rs7873690, rs684523, rs1619249, rs263156) explains the variation in tragus size. The genotype change from CC to TT in rs17023457, which is 3.175 times more likely (p- value=0.044), from GG to TT in rs868157 which is 5.235 times (p-value=0.041), from TT to CT in rs7873690 which is 2.452 times (p value=0.041), from TT to CT in rs684523 which is 1.922 times (p-value=0.038), from CC to TT in rs1619249, it is 5.609 times (p-value= 0.028) more likely to get prominent tragus. The genotype change from CC to CT in rs7428 making it 0.505 times (p-value=0.032), from CC to TT in rs7428 making it 0.432 times (p-value=0.009), from AA to AC in rs263156 which is 0.505 times (p-value=0.049), less likely to get prominent tragus.

Subjects were 0.669 times less likely to have under folded superior helix rolling when compared with overfolded and 7.691 times more likely to get partially folded helix rolling as compared with overfolded superior helix rolling. The highest statistical significance was obtained for two SNP (rs13397666, rs7567615) explains the variation in superior helix rolling. The genotype changed from GG to AG in rs13397666 it is 2.24 times (p- value=0.041).

Subjects were 1.386 times more likely to get under folded posterior helix rolling when compared with overfolded posterior helix rolling and 5.716 times more likely to have partially folded helix rolling when compared with overfolded posterior helix rolling. The highest statistical significance was obtained for four SNPs (rs7428, rs684523, rs1619249, rs263156) explains the variation in posterior helix rolling. The genotype changed from CC to CT in rs7428m which is 0.505 times (p-value=0.032), from CC to TT in rs7428 which is 0.432 times (p-value=0.021), from AA to AC in rs26315, which is 0.505 times (p-value= 0.049) , less likely to get overfolded posterior helix rolling. The genotype changed from TT to CT in rs684523 making it 1.922 times (p- value=0.038), from CC to TT in rs1619249 making it 5.609 times (p value=0.028), more likely to get prominent posterior helix rolling.

Subjects were 0.497 times less likely to get under folded antihelix folding when compared with overfolded ones and 4.806 times more likely to get partially folded antihelix folding when compared with overfolded antihelix folding. The highest statistical significance was obtained for two SNPs (2080401, rs260674) explains the variation with antihelix folding. The genotype change from CC to AC in rs2080401 makes it 2.496 times (p-value=0.016) more likely to have overfolded antihelix folding. The genotype change from GG to AA in SNP rs260674 which is 0.190 times (p value=0.041) less likely to get prominent antihelix folding.

Subject were 0.001 times less likely to have flat antihelix superior crus when compared with extended antihelix superior crus and 0.011 times less likely to have intermediate antihelix superior crus when compared with extended helix superior crus. The highest statistical significance was obtained for seven SNPs (17023457, rs7567615, rs1960918, rs9866054, rs10192049, rs13427222, rs1878495) explains variation in antihelix superior crus. The genotype changed from AA to GG in rs7567615 which is 2.182 times (p value= 0.031), from CC to TT in rs1960918 which is 3.420 times (p-value=0.005) more likely to have extended antihelix superior crus. The genotype change from CC to CT in rs17023457 which is 0.232 times (p- value=0.027), from AA to GG in rs9866054 making it 0.393 times (p value= 0.048) , from GG to AG in SNP rs1342722 making it 0.260 time (p-value=0.238), from GG to AA in rs10192049 which is 0.391 times ( p-value= 0.028) , from GG to AA in rs13427222 which is 0.267 times (p-value= 0.037), from AA to CC in rs1878495 which is 0.444 times (p-value=0.048), less likely to get prominent antihelix superior crus.

Subjects were 6 times more likely with an absent Darwin Tubercle when compared with prominent ones and 1.076 times more likely to get a degree of tubercle when compared with prominent ones. The highest statistical significance was obtained for two SNPs (rs13397666, rs260674) explains the variations in Darwin Tubercle. The genotype changed from GG to AG which is rs1339766, it is 3.471 times (p- value=0.049) more likely to have prominent Darwin Tubercle. The genotype change from GG to AA in rs260674 which is 0.125 times (p value=0.029) less likely to get prominent Darwin Tubercle.

Subjects were 0.843 times less likely to have small crus helix when compared with Extended crus helix and 6.402 times more likely (p-value=0.048) to have prominent crus helix when compared with extended crus helix. The highest statistical significance was obtained for three SNPs (rs7428, rs2080401, rs7873690) explains the variation with crus helix expression. The genotype change from CC to CT in rs7428 which is 0.528 times (p- value=0.036), from CC to AA in rs2080401 which is 0.510 times (p value=0.048), from TT to CC in rs78736900 which is 0.476 times (p-value= 0.048), less likely to get extended crus helix expression.

Subjects were 0.266 times less likely (p-value =0.044) to have small ear protrusion when compare to large ear protrusion and 2.366 times more likely (p-value=0.618) to have partial protrusions when compared with large protrusion. The highest statistical significance was obtained for two SNPs (rs263156, rs1878495) explain variation with ear protrusion). The genotype changed from AA to CC in rs1878495, it is 0.474 times (p value=0.041) ,from AA to CC in rs263156 it is 0.474 times (p-value=0.041) less likely to get large protrusion.
